# Aurora-A/SOX8/FOXK1 signaling axis promotes chemoresistance via suppression of cell senescence and induction of glucose metabolism in ovarian cancer organoids and cells

**DOI:** 10.7150/thno.43811

**Published:** 2020-05-25

**Authors:** Huizhen Sun, Husheng Wang, Xue Wang, Yoichi Aoki, Xinjing Wang, Yufei Yang, Xi Cheng, Ziliang Wang, Xipeng Wang

**Affiliations:** 1 Department of Gynecology and Obstetrics, Xinhua Hospital Affiliated to Shanghai Jiaotong University School of Medicine, Shanghai 200092, China.; 2 Department of Gynecologic Oncology, Cancer Institute Hospital, Tokyo, Japan.; 3 Department of Gynecological Oncology, Fudan University Shanghai Cancer Center, Shanghai 200032, China.

**Keywords:** Aurora-A, SOX8, PDOs, Chemoresistance, Ovarian cancer

## Abstract

**Rationale:** Cisplatin derivatives are first-line chemotherapeutic agents for epithelial ovarian cancer. However, chemoresistance remains a major hurdle for successful therapy and the underlying molecular mechanisms are poorly understood at present.

**Methods:** RNA sequencing of organoids (PDO) established from cisplatin-sensitive and -resistant ovarian cancer tissue samples was performed. Glucose metabolism, cell senescence, and chemosensitivity properties were subsequently examined. Immunoprecipitation, mass spectrometry, Fӧrster resonance energy transfer-fluorescence lifetime imaging (FRET-FLIM), luciferase reporter assay, ChIP and animal experiments were conducted to gain insights into the specific functions and mechanisms of action of the serine/threonine kinase, Aurora-A, in ovarian cancer.

**Results:** Aurora-A levels were significantly enhanced in cisplatin-resistant PDO. Furthermore, Aurora-A promoted chemoresistance through suppression of cell senescence and induction of glucose metabolism in ovarian cancer organoids and cells. Mechanistically, Aurora-A bound directly to the transcription factor sex determining region Y-box 8 (SOX8) and phosphorylated the Ser327 site, in turn, regulating genes related to cell senescence and glycolysis, including hTERT, P16, LDHA and HK2, through enhancement of forkhead-box k1 (FOXK1) expression.

**Conclusions:** Aurora-A regulates cell senescence and glucose metabolism to induce cisplatin resistance by participating in the SOX8/FOXK1 signaling axis in ovarian cancer. Our collective findings highlight a novel mechanism of cisplatin resistance and present potential therapeutic targets to overcome chemoresistance in ovarian cancer.

## Introduction

Ovarian cancer remains the most lethal gynecologic malignancy, with ~22,000 new cases and 14,000 deaths estimated in 2018 in the US alone 1. Despite substantial progress in diagnosis and therapy over the years, the overall 5-year survival rate remains extremely low at 25-35% 2 owing to the considerable difficulty in detecting the disease at the early stages 3 and common development of resistance to cisplatin- or carboplatin-based chemotherapy 4. As a result, fewer than 15% of patients respond to further treatment 4, leading to an average progression-free survival (PFS) of 3-4 months and median overall survival (OS) of 9-12 months 5. The molecules and genetic factors that drive the development of chemoresistance are yet to be established, which presents a major obstacle in preventing and predicting clinical chemoresistance.

Various cell lines are available for modeling chemoresistance of ovarian cancer due to the characteristics of easy operation and stable culture. However, cancer cell clusters in tumor tissues are heterogeneous, which is important for the evolution-associated tumor microenvironment (TME) and chemoresistance. Since ovarian cancer tissues comprise a wide spectrum of distinct molecules and clinical entities 6, cell lines derived from single clones are unable to effectively model the TME and organ functions. An organoid is a three-dimensional multicellular structure with microanatomical features of its source organs 7. More recently, patient-derived organoids (PDO) representing tumor-relevant physiology and pathology have been successfully applied to achieve rapid assaying of phenotype-genotype correlations and drug sensitivity in many tumor types 8-10, offering an exceptional model to study cancer chemoresistance.

The serine/threonine kinase, Aurora-A, is a member of the Aurora kinase family reported to induce centrosome amplification, spindle formation, chromosomal instability and transformation in mammalian cells 11,12. The protein is frequently overexpressed and/or amplified in various cancer types, including breast cancer 13, pancreatic cancer 14 and ovarian cancer 11, and its upregulation was shown to be associated with drug resistance and poorer prognosis 15-17. Conversely, interference with Aurora-A expression is reported to sensitize tumor cells to antineoplastic treatments 18,19, highlighting its potential utility as a promising chemotherapeutic target 20-23.

In this study, we established organoids from both cisplatin-sensitive and -resistant ovarian cancer tissues. RNA sequencing revealed significant upregulation of Aurora-A in cisplatin-induced resistant PDOs. Furthermore, Aurora-A promoted cisplatin resistance via regulation of cell senescence and glucose metabolism in PDOs, cells and tumor-transplanted mice. Data from mechanistic analyses showed that Aurora-A bound directly to the transcription factor sex determining region Y-box 8 (SOX8) and phosphorylated its Ser327 site, thereby activating forkhead-box k1 (FOXK1) with consequent effects on cell senescence, glycolysis, and chemoresistance. Our collective findings disclose a previously unrecognized mechanism of chemoresistance in ovarian cancer, which provides novel therapeutic targets that may be effective in preventing tumor chemoresistance and progression.

## Results

### Cisplatin resistance is closely associated with cell senescence and glucose metabolism in organoids of ovarian cancer

**Figure 1:**
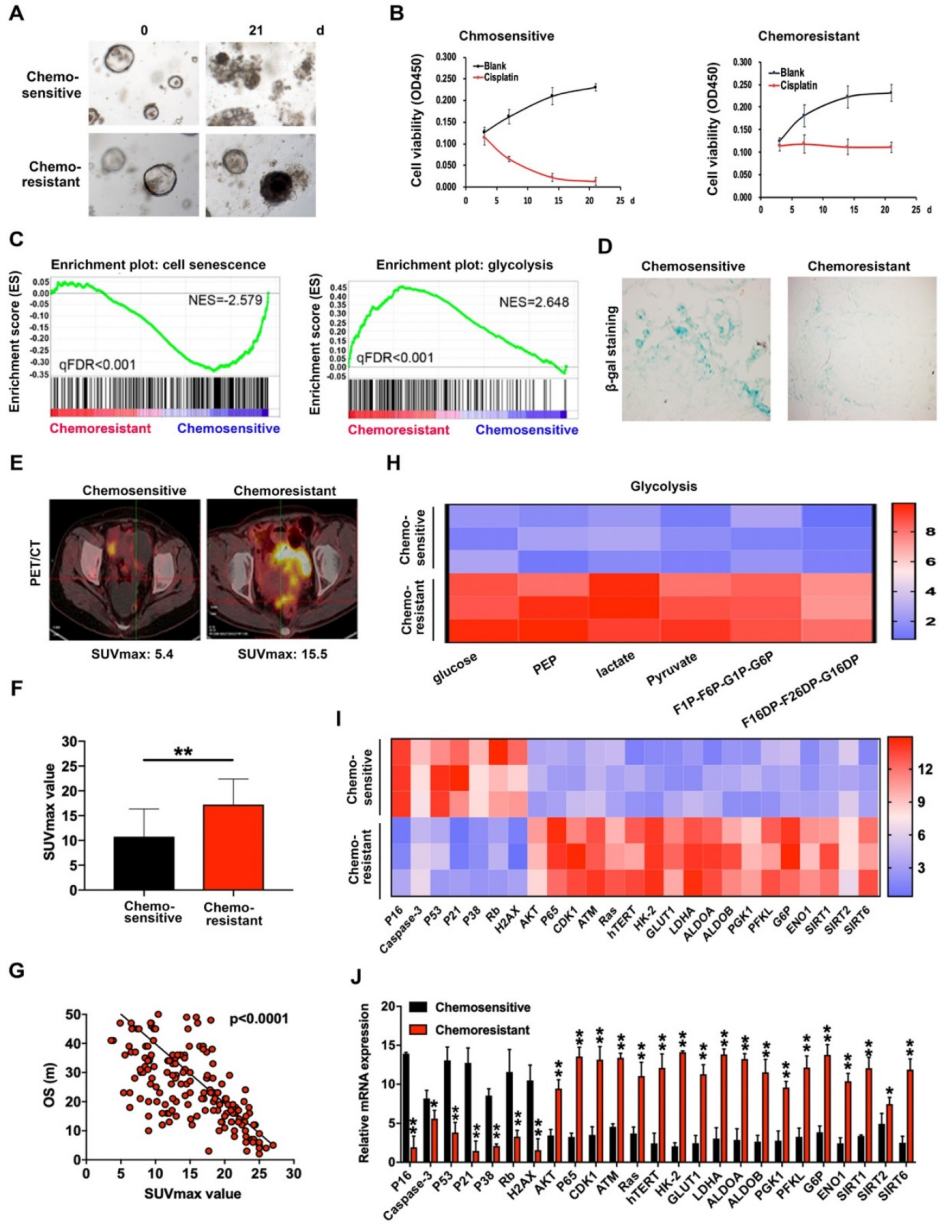
** Cisplatin-resistance is closely associated with cell senescence and glucose metabolism in organoids of ovarian cancer. (A)** Images of cisplatin-sensitive and -resistant PDOs of ovarian cancer with 3ug/L cisplatin treatment on days 0 and 21. **(B)** Cell viability assay of PDOs treated with 3 µg/L cisplatin in different time intervals. **(C)** GSEA analysis was performed using cisplatin-sensitive and cisplatin-resistant PDOs of ovarian cancer. The signature was defined by genes with significant expression changes. **(D)** Representative images of β-galactosidase staining in cisplatin-sensitive and cisplatin-resistant ovarian cancer tissues. **(E and F)** The relationship between SUVmax value of PET/CT image and chemosensitivity in ovarian cancer patients. SUVmax value of PET/CT scan in 100 cisplatin-sensitive and 100 cisplatin-resistant ovarian cancer patients were analyzed. **(G)** The association between SUVmax value of PET/CT image and overall survival in ovarian cancer patients. SUVmax value of PET/CT scan in 200 ovarian cancer patients were analyzed. **(H)** Metabolites in glycolysis pathway. **(I) and (J)** Heat map (**I**) and histogram (**J**) of qRT-PCR analysis of indicated genes in cisplatin-sensitive and cisplatin-resistant ovarian cancer organoids. Data were shown as mean ± SD. Significance was calculated using the Student t test. *P < 0.05, **P < 0.01.

To explore the potential mechanisms underlying ovarian cancer chemoresistance, we established PDOs using cisplatin-sensitive and -resistant ovarian cancer tissues and verified their chemosensitivity to cisplatin (**Figure 1A-B**). RNA sequencing was employed to compare expression of chemosensitivity-related genes in 4 cisplatin-sensitive and 6 cisplatin-resistant PDOs. Notably, levels of a number of important regulatory genes involved in cell senescence were decreased while those of several genes involved in glycolysis were significantly increased (**Figure 1C**), as confirmed with SA-β-gal (**Figure 1D**) and patient PET/CT scans (**Figure 1E**). The SUV_max_ values of ovarian cancer patients subjected to PET/CT were additionally assessed. The average value for cisplatin-resistant patients was 17.23 while that for cisplatin-sensitive patients was 10.75 (**Figure 1F**). Overall, high SUV_max_ values associated with poorer overall survival (**Figure 1G**). Data from the glucose metabolism assay showed an obvious increase in metabolites of glycolysis in cisplatin-resistant PDOs (**Figure 1H**). Subsequent qRT-PCR performed to detect mRNA expression of cisplatin-sensitive and -resistant PDOs revealed that levels of several genes inducing cell senescence, such as p53, p16 and p21, were decreased in cisplatin-resistant PDOs while those participating in glycolysis, such as HK2, GLUT1 and LDHA, were significantly increased (**Figure 1I-J**), clearly indicating critical involvement of cell senescence and glycolysis in chemoresistance of ovarian cancer.

### Chemoresistance of ovarian cancer was induced by Aurora-A high expression

**Figure 2:**
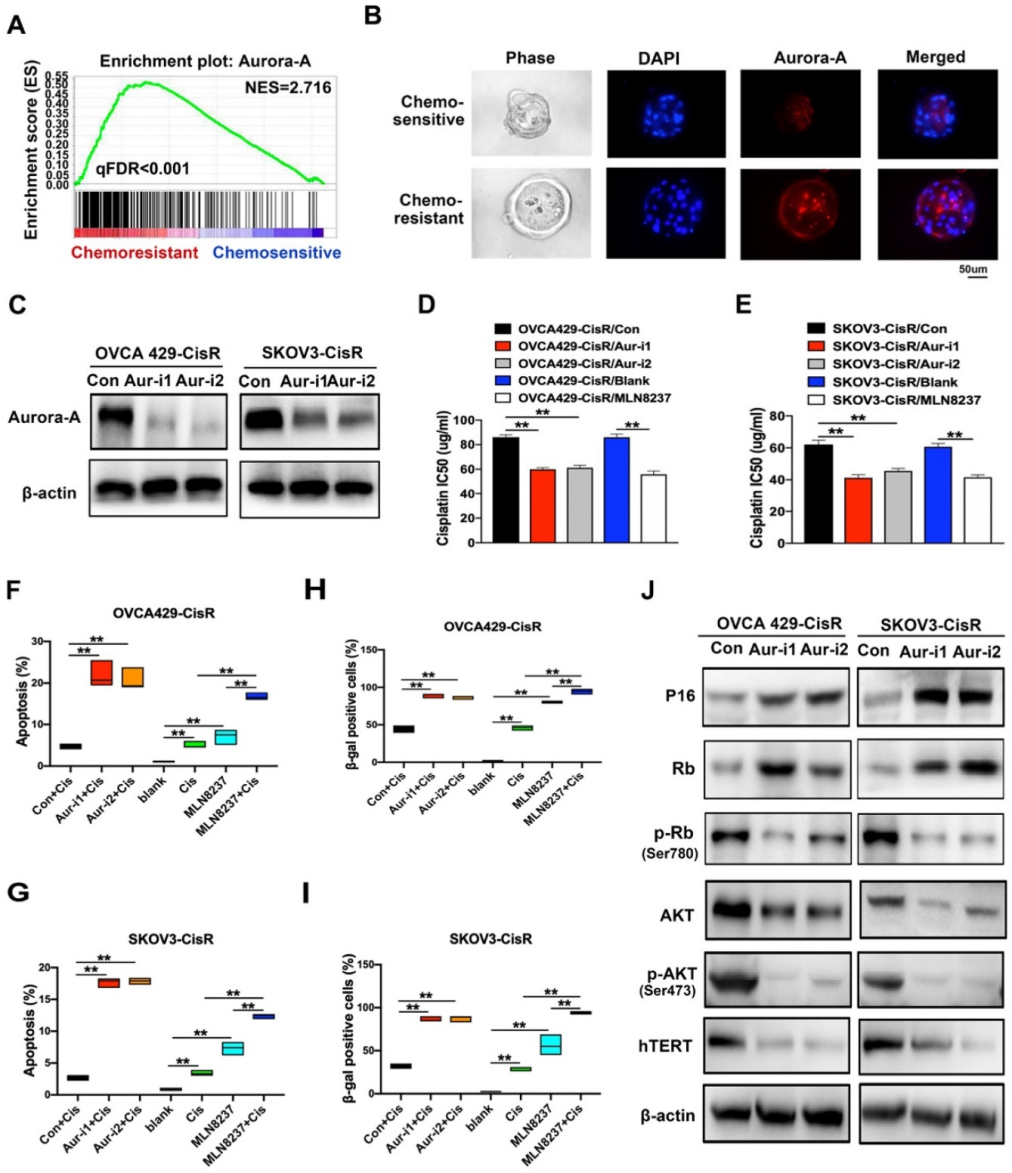
** Aurora-A regulates chemoresistance and cell senescence in ovarian cancer. (A)** GSEA analysis was performed using cisplatin-sensitive and cisplatin-resistant PDOs of ovarian cancer. The signature was defined by genes with significant expression changes. **(B)** Immunofluorescence assay detected the relation of Aurora-A and chemosensitivity in PDOs of ovarian cancer. **(C)** Aurora-A knockdown cell lines were established by immunoblotting. **(D and E)** Values of IC50 of cisplatin. Aurora-A silencing cells, 0.5 nM MLN8237-treated cells and their controls were treated with cisplatin in different concentrations for 48h. **P < 0.01. **(F) and (G)** Percentage of apoptotic cells. Except the blank group, cells were treated with 5 µg/mL cisplatin, 0.5nM MLN8237, or 5 µg/mL cisplatin plus 0.5 nM MLN8237 for 48 h, respectively. Apoptosis was measured by flow cytometry in cells stained with annexin V and propidium iodide. **P < 0.01. **(H and I)** β-galactosidase staining. **P < 0.01. Cells were treated with the same method as Figure 2F-G. **(J)** Immunoblotting analysis of proteins associated with the cell senescence and apoptosis.

To distinguish chemoresistance-associated genes, RNA sequencing assays were conducted, which led to the identification of Aurora-A as a potential crucial regulator in cisplatin-resistant PDOs (**Figure 2A**). Data from immunofluorescence analyses showed markedly higher Aurora-A expression in cisplatin-resistant PDOs relative to the control group (**Figure 2B**), which was further confirmed via qRT-PCR of tumor tissues (**Figure S1A**). To investigate the functions and mechanisms of action of Aurora-A in chemoresistance, we established ovarian cancer cell lines, OVCA429 and SKOV3, overexpressing Aurora-A. Interestingly, upregulation of Aurora-A significantly attenuated sensitivity of cells to cisplatin (F**igure S1B-C**). We subsequently generated cisplatin-resistant cell lines, OVCA429-CisR and SKOV3-CisR (**Figure S1D**) for examination of Aurora-A expression. Immunoblot analyses showed significant expression of Aurora-A in both OVCA429-CisR and SKOV3-CisR cell lines (**Figure S1E**). Subcellular localization analysis verified that cytoplastic Aurora-A levels were significantly increased in chemoresistant ovarian cancer cells while nuclear Aurora-A was only slightly affected (**Figure S1F-H**). The effects of Aurora-A silencing on cell viability and apoptosis were further examined by transfection of two shRNA vectors of Aurora-A into OVCA429-CisR and SKOV3-CisR cell lines (**Figure 2C**). Notably, knockdown of Aurora-A significantly suppressed viability in ovarian cancer cells, as observed from CCK-8 experiments (**Figure S1I**). Next, we examined whether Aurora-A expression and activity are associated with cisplatin resistance in ovarian cancer cells. To this end, MLN8237 was used to selectively inhibit Aurora-A activity and the effects were confirmed via immunoblotting of p-Aurora-A (Thr288) (**Figure S1J**). Compared with the corresponding control cells, the half-maximal inhibitory concentration (IC_50_) of cisplatin was decreased in OVCA429-CisR cells by 30.41%, 28.88% and 35.11% after Aur-i1, Aur-i2 and MLN8237 treatment; IC_50_ was decreased in SKOV3-CisR cells by 33.65%, 26.60% and 33.08% after Aur-i1, Aur-i2 and MLN8237 treatment compared with their controls (**Figure 2D-E**).

By Flow Cytometry we found that the number of apoptotic cells induced by 5ug/mL cisplatin for 48h in OVCA429-CisR was increased by 17.27%, 16.09% and 12.07%, respectively, after Aur-i1, Aur-i2 and MLN8237 treatment; in SKOV3-CisR the number of apoptotic cells was increased by 14.93%, 15.13%, and 9.63% after Aur-i1, Aur-i2 and MLN8237 treatment compared with their controls (**Figure 2F-G and Figure S1K**). And in comparison with MLN8237 alone, combination treatment with cisplatin increased apoptosis by 9.59% in OVCA429/CisR cells and 5.07% in SKOV3-CisR cells. Moreover, wild-type Aurora-A overexpression led to reversal of chemosensitivity caused by Aurora-A silencing (**Figure S2A-F**), clearly signifying a critical role of this protein in mediating chemoresistance of ovarian cancer cells.

### Aurora-A suppresses cell senescence and promotes glycolysis in ovarian cancer cells

Since chemoresistance appears to be associated with low senescence in PDOs of ovarian cancer, senescent ovarian cancer cells were established for further study. Chemoresistance was effectively reversed in the senescent group, compared with the control (**Figure S3A-C**). Moreover, SA-β-gal activity was increased in Aurora-A knockdown cells after 15 passages (**Figure S3D**) or treatment with cisplatin (**Figure 2H-I and Figure S3E**). Immunofluorescence and immunoblotting experiments demonstrated that Aurora-A knockdown increased apoptosis and senescence-associated proteins, such as P16, concomitant with a significant decrease in hTERT (**Figures 2J and S3F**).

**Figure 3:**
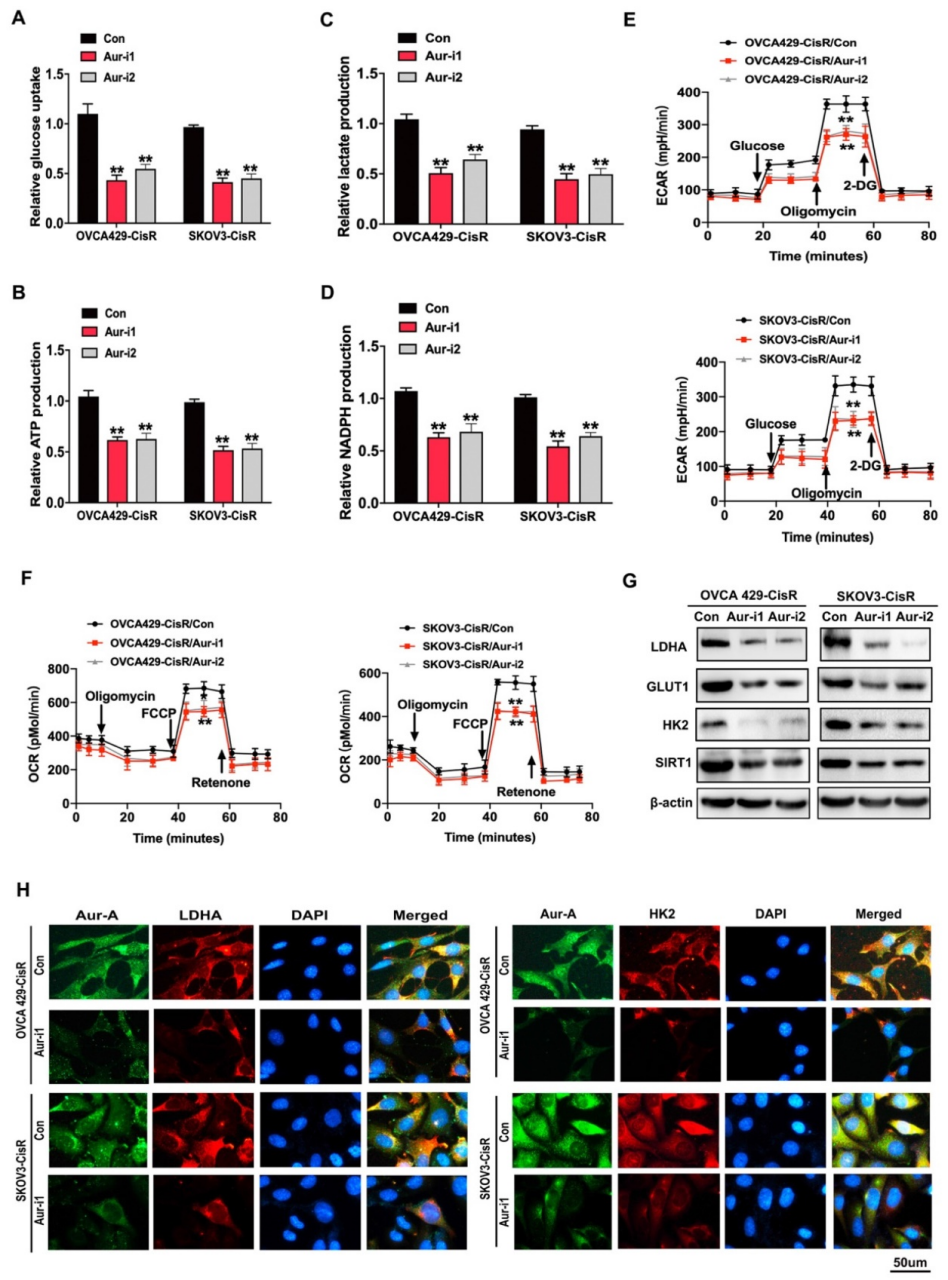
** Aurora-A modulated the glucose metabolism in ovarian cancer cells. (A-D)** Glucose uptake (**A**), ATP (**B**), lactate (**C**) and NADPH (**D**) production were determined as described in Methods. “CON” was the cells transfected with scrambled shRNA expression vector. Data shown were mean ± SD of triplicate measurements repeated 3 times with similar results. Statistical significance was assessed by two-tailed Student's t test. **(E and F)** ECAR (**E**) and OCR (**F**) were determined as described in Methods. **(G)** Immunoblotting analysis of glycolytic gene expression in Aurora-A knockdown ovarian cancers and the controls. **(H)** Immunofluorescence assay detected the relationship between Aurora-A and glycolysis associated proteins.

Since chemoresistance is associated with high glycolysis in PDOs of ovarian cancer, we investigated this association in ovarian cancer cells. As expected, chemoresistant ovarian cancer cells exhibited higher glycolysis phenotypes, compared to their parental cells (**Figure S4A-F**). To validate these results, chemoresistant ovarian cancer cells were treated with the glycolysis inhibitor 2-DG (5 mM for 48 h), which led to significant reversal of cisplatin resistance (**Figure S4G-H**). Subsequently, cellular glycolysis assays were conducted to examine the effects of Aurora-A on glucose metabolism in ovarian cancer cells. Glycolytic phenotypes were clearly increased under conditions of Aurora-A upregulation (**Figure S4I-N**) while Aurora-A silencing inhibited glycolysis to a significant extent (**Figure 3A-F**).

Immunoblotting and immunofluorescence experiments further revealed that Aurora-A knockdown led to reduced expression of glycolysis-related proteins, such as LDHA and HK2, in OVCA429-CisR and SKOV3-CisR cell lines (**Figure 3G-H**). The collective results indicate that Aurora-A inhibition exerts anticancer effects through promoting cell senescence and inhibiting glucose metabolism.

### Aurora-A regulates the SOX8/FOXK1 pathway to affect cell senescence and glycolysis in cisplatin-resistant ovarian cancer cells

**Figure 4:**
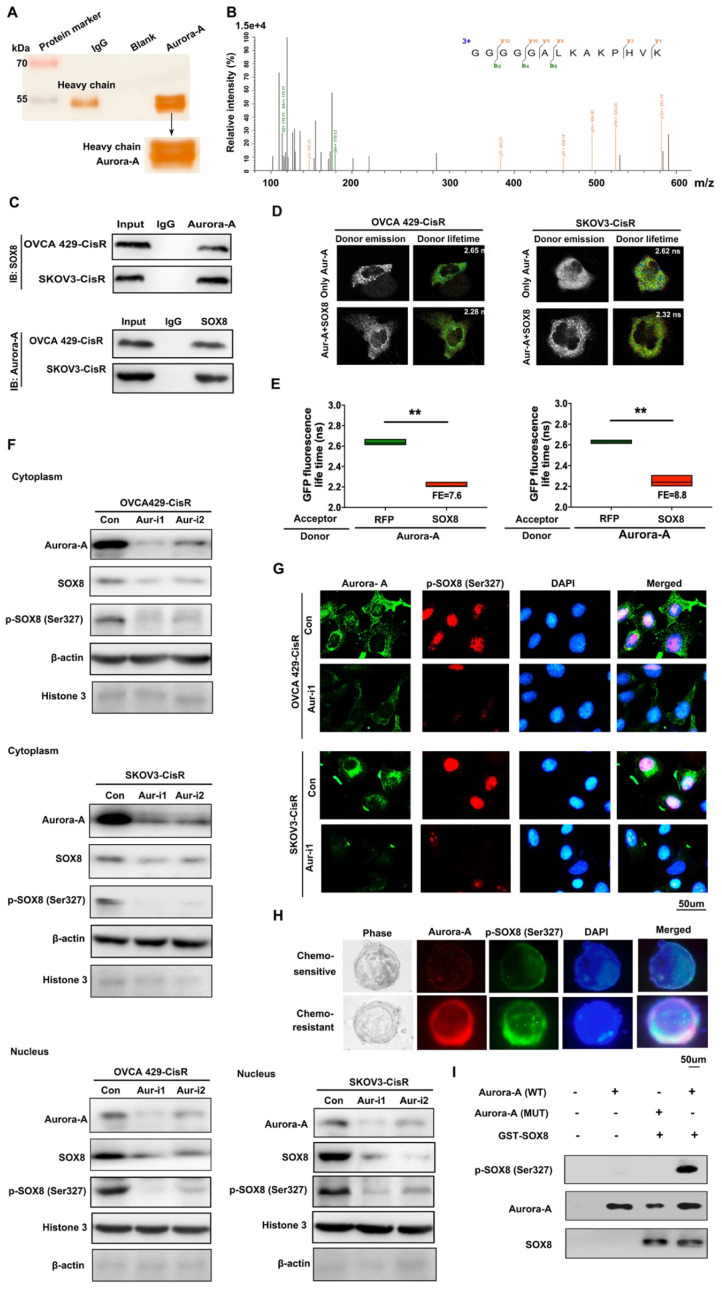
** Aurora-A combined with SOX8 directly and activated it by phosphorylation. (A)** Cellular extracts from SKOV3-CisR cells were immunopurified with anti-Aurora-A affinity columns and eluted with Aurora-A peptide. The eluates were resolved by SDS-PAGE and silver stained. **(B)** Mass spectrometry analysis of the SDS-PAGE above. The detected peptides spectra of SOX8 were listed. **(C)** Co-IP analysis demonstrated an interaction between Aurora-A and SOX8 protein in OVCA429-CisR and SKOV3-CisR cell lines. **(D and E)** Interaction between Aurora-A and SOX8 confirmed by FRET-FLIM upon transient co-expression. FE, FRET efficiency. **P < 0.01. **(F)** Aurora-A silencing significantly changed the distribution and expression of SOX8 and p-SOX8 (Ser327) in cytoplasm and nucleus of ovarian cancer cells. **(G and H)** Immunofluorescence assay detected the relation of Aurora-A and p-SOX8 (Ser327) in ovarian cancer cell lines (**G**) and PDOs (**H**). **(I)** In vitro kinase assay to detect the phosphorylation of recombinant GST-SOX8 protein by wild-type or mutant Aurora-A coprecipitates.

To identify the potential targets of Aurora-A in ovarian cancer cells, immunoprecipitation (IP) assay and mass spectrometry analyses were performed. Interestingly, the transcription factor SOX8 was identified as a directly interacting molecule with Aurora-A (**Figure 4A-B**), which was further confirmed via co-IP (**Figure 4C**) and Fӧrster resonance energy transfer-fluorescence lifetime imaging (FRET-FLIM; **Figure 4D-E**). Our data suggest that SOX8 is a potential target of Aurora-A in ovarian cancer.

In view of the finding that Aurora-A is a serine/threonine kinase that functions by phosphorylating proteins, IP and mass spectrometry analyses were conducted, which led to the identification of Ser327 as the phosphorylation site. Immunoblot experiments further confirmed Aurora-A-induced phosphorylation of SOX8 at Ser327. Interestingly, Aurora-A silencing led to reduced accumulation of SOX8 and p-SOX8 (Ser327) proteins mainly in the nucleus, with little effects on the levels of the corresponding cytoplasmic proteins (Ser327; **Figure 4F**). Next, we examined the regulatory effect of Aurora-A on p-SOX8 via immunofluorescence in both cell lines and PDOs (**Figure 4G-H**). Moreover, data from *in vitro* kinase assays consistently showed that recombinant GST-SOX8 expressed and purified from *Escherichia coli* was phosphorylated at Ser327 by wild-type Aurora-A coprecipitates (**Figure 4I**). Finally, we mutated the phosphorylation site in chemoresistant cells and performed immunoblot assay to test the nuclear SOX8 expression level. The results showed that the expression of SOX8 in nuclei was reduced significantly, and functional experiments suggested that the mutant-SOX8 could not rescue the chemosensitivity induced by Aurora-A silencing (**Figure S5A-C**).

To further determine whether SOX8 is a critical target gene of Aurora-A, we performed a rescue experiment with overexpression of SOX8 in Aurora-A silencing cells (**Figure S5D**) and examined the impacts on cell viability, cisplatin sensitivity, senescence and glycolysis. In both OVCA429-CisR and SKOV3-CisR cell lines, SOX8 overexpression partially reversed the changes in cell viability caused by Aurora-A silencing (**Figure S5G**). In addition, Aurora-A silencing-mediated effects on cisplatin sensitivity, senescence, metabolites and glucose consumption were significantly reversed (**Figure S5H-J and S6A-F**). Data from qRT-PCR analyses additionally showed that SOX8 transfection partially reversed the changes in cell senescence and glycolysis-associated proteins (**Figure S5K, 6G**). In the luciferase reporter assay, SOX8 transfection led to significant inhibition of P16 promoter activity, increase in hTERT promoter activity (**Figure S5L-M**), and increase in glycolysis-associated HK2 and LDHA promoter activities (**Figure S6H-I**).

**Figure 5:**
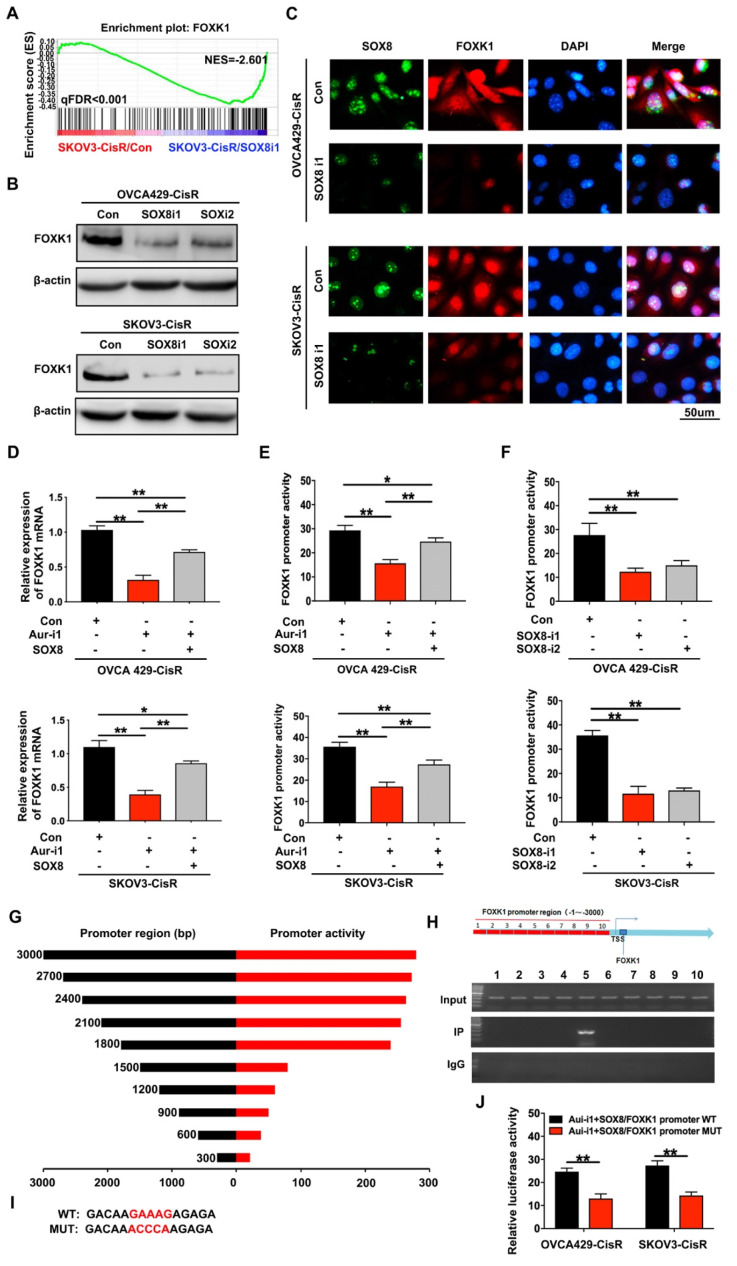
** FOXK1 is the downstream targeting molecule of SOX8. (A)** GSEA analysis was performed using SOX8 knockdown cell line and the control (SKOV3-CisR/SOX8-i1 and SKOV3-CisR/Con). The signature was defined by genes with significant expression changes. **(B and C)** Immunoblotting and immunofluorescence assay detected the relation of SOX8 and FOXK1 in ovarian cancer cells. **(D)** FOXK1 mRNA expression level in Aurora-A knockdown ovarian cancer cell lines and SOX8 transfected cell lines in rescue experiment. **(E)** FOXK1 promoter activity affected by Aurora-A knockdown and SOX8 overexpression.** (F)** FOXK1 promoter activity affected by SOX8 silencing.** (G)** The region of SOX8 binding sites within the FOXK1 promoter. **(H)** ChIP results of the binding of SOX8 to the promoter of FOXK1.** (I)** The map of SOX8 binding sits in the promoter region of FOXK1. **(J)** Luciferase reporter assay was used for the detection of mutant sites in the promoter region of FOXK1. **P < 0.01.

To elucidate the mechanistic involvement of SOX8, we transfected two different shRNA vectors of SOX8 into OVCA429-CisR and SKOV3-CisR cell lines (**Figure S5E**). RNA sequencing data showed that SOX8 knockdown significantly inhibited FOXK1 expression (**Figure 5A**), which was confirmed in cell lines via immunoblotting and immunofluorescence (**Figure 5B-C**). qRT-PCR results showed downregulation of FOXK1 mRNA upon knockdown of Aurora-A in both OVCA429-CisR and SKOV3-CisR cells. However, following transfection of SOX8 cDNA, FOXK1 expression was partially rescued (**Figure 5D**). Furthermore, a luciferase reporter assay was performed with a FOXK1 promoter luciferase reporter plasmid to determine mechanistic associations among Aurora-A, SOX8 and FOXK1. First, we transfected FOXK1 promoter plasmids into OVCA429-CisR and SKOV3-CisR cell lines with Aurora-A knockdown and overexpression of SOX8. Compared with control groups, Aurora-A silencing led to significant inhibition of FOXK1 promoter activity. However, when cells were transfected with SOX8 cDNA, FOXK1 promoter activity was partially rescued (**Figure 5E**). In OVCA429-CisR and SKOV3-CisR cells depleted of SOX8, FOXK1 promoter activity was markedly decreased (**Figure 5F**). To confirm the precise SOX8 binding site within the FOXK1 promoter, we cloned promoter fragments of different lengths for analysis of *cis*-acting elements (**Figure 5G**). ChIP analysis of SKOV3-CisR cell lines led to the identification of a single binding site located upstream of the transcription start site (**Figure 5H**). To further establish the binding site of SOX8, we individually mutated it and repeated the luciferase assay (**Figure 5I**). Our results showed that mutation of the binding site alone resulted in abrogation of luciferase activity (**Figure 5J**).

To determine the potential functions and mechanisms of action of FOXK1 in chemoresistance, senescence and glycolysis in ovarian cancer cells, OVCA429-CisR/Aur-i1/SOX8 and SKOV3-CisR/Aur-i1/SOX8 cell lines with FOXK1 knockdown were generated (**Figure S5F**). In both cell lines, FOXK1 silencing significantly reversed the changes in cell viability induced by SOX8 transfection (**Figure S5G**). In addition, FOXK1 knockdown significantly reversed SOX8-mediated cisplatin sensitivity, cell senescence, metabolites and glucose consumption (**Figure S 5H-J and S6A-F**). Similar results were observed with qRT-PCR, whereby FOXK1 knockdown partially reversed the alterations in cell senescence and glycolysis-associated proteins (**Figure S5K and S6G**). In the luciferase reporter assay, FOXK1 silencing partially increased the changes in P16 promoter activity, and suppressed the changes in hTERT, HK2 and LDHA promoter activity induced by SOX8 transfection (**Figure S5L-M and S6H-I**). Furthermore, ChIP assay was performed to verify the transcriptional regulation (**Figure S5N and S6J**).

The above results clearly indicate that Aurora-A contributes to cisplatin resistance by inhibiting cell senescence and promoting glycolysis through regulation of the SOX8/FOXK1 signaling pathway in ovarian cancer.

### Aurora-A knockdown inhibits the progression of ovarian cancer and sensitizes cancer cells response to cisplatin *in vivo*

**Figure 6:**
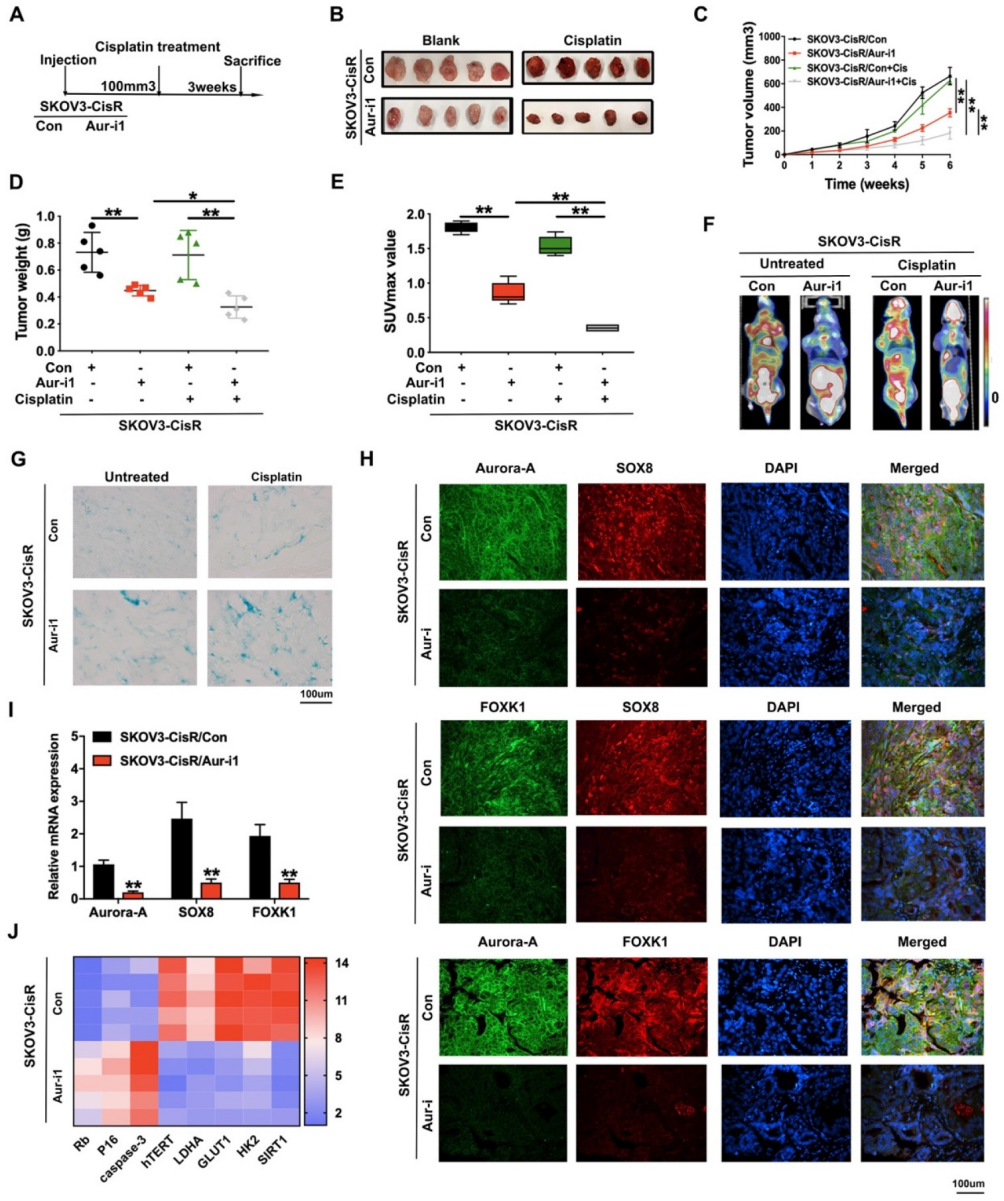
** Aurora-A knockdown inhibited the progression of ovarian cancer and sensitized cancer cells response to cisplatin *in vivo*. (A)** Flow chart of the *in vivo* experiment. **(B)** Representative image of nude mice bearing tumors formed by SKOV3-CisR/Aur-i1 and their control cells with cisplatin treated or untreated. **(C)** Growth curve of the xenograft tumors burdened in mice. **(D)** The average tumor weight of nude mice.** (E)** Average SUVmax values of nude mice bearing tumors.** (F)** Representative image of PET-CT, which was used for the detection of glucose uptake. **(G)** Representative image of β-galactosidase staining of the nude mice tumor tissues with or without cisplatin treatment. **(H)** Immunofluorescence assay detected the relation among Aurora-A, SOX8, and FOXK1 in the nude mice tumor tissues with cisplatin treatment.** (I)** The mRNA expression levels of Aurora-A, SOX8, and FOXK1 were tested by qRT-PCR assay in tumors* in vivo.* Error bars: 95% Cis; **P < 0.01. **(J)** Heat map showed the gene expression associated with cell senescence and glycolysis in the nude mice tumor tissues with cisplatin treatment.

Tumor promotion and chemoresistance induction functions of Aurora-A *in vivo* were subsequently examined. Firstly, SKOV3-CisR cells with either Aurora-A knockdown or harboring empty vector were injected into flanks of nude mice and tumor sizes were carefully observed. Mice were treated with cisplatin on alternate days when tumor volumes reached 100 mm^3^ (**Figure 6A**). As shown in **Figure 6B-D**, Aurora-A depletion led to a decrease in the speed of tumor growth and overall tumor weight *in vivo*. Aurora-A silencing in combination with cisplatin treatment induced a further reduction in tumor volume and weight, compared with cisplatin alone. In PET-CT analysis, silencing of Aurora-A strongly suppressed glucose uptake of xenograft cells *in vivo* and resulted in lower SUV_max_ values (**Figure 6E-F**). SA-β-gal staining of cisplatin-treated xenograft tissues disclosed that Aurora-A knockdown increased cell senescence (**Figure 6G**). Immunofluorescence and qRT-PCR analyses were further employed to validate the relationships among Aurora-A, SOX8 and FOXK1 in the cisplatin treatment groups. Our data showed that Aurora-A knockdown reduced SOX8 and FOXK1 expression in tumors *in vivo* (**Figure 6H-I**), with a positive association between SOX8 and FOXK1 expression patterns. Interestingly, Aurora-A silencing indirectly restrained SOX8 transcription, which may be induced by the downregulation of oncogenic transcription factor c-Myc in Aurora-A depleted group (**Figure S7A**). Furthermore, SOX8 transcription was effectively rescued by c-Myc overexpression, which was verified via RT-PCR and dual luciferase reporter assay (**Figure S7B-C**). In addition, immunofluorescence analyses to determine the associations between Aurora-A and essential proteins involved in cell senescence and glycolysis in xenograft tissues revealed that Aurora-A knockdown after cisplatin treatment reduced hTERT, HK2 and LDHA and increased P16 expression (**Figure S7D-G**). Subsequently, mRNA was extracted from transplanted murine tumors and RT-qPCR was performed to validate the involvement of Aurora-A in cell senescence and glycolysis. In accordance with the earlier results obtained with cell lines, Aurora-A depletion enhanced the expression of specific apoptosis- and cell senescence-associated proteins, such as P16 and Rb, and suppressed hTERT as well as proteins involved in glycolysis, such as LDHA, GLUT1, HK2, and SIRT1 (**Figure 6J**).

### Expression of Aurora-A, SOX8, and FOXK1 is associated with poor survival in ovarian cancer patients

**Figure 7:**
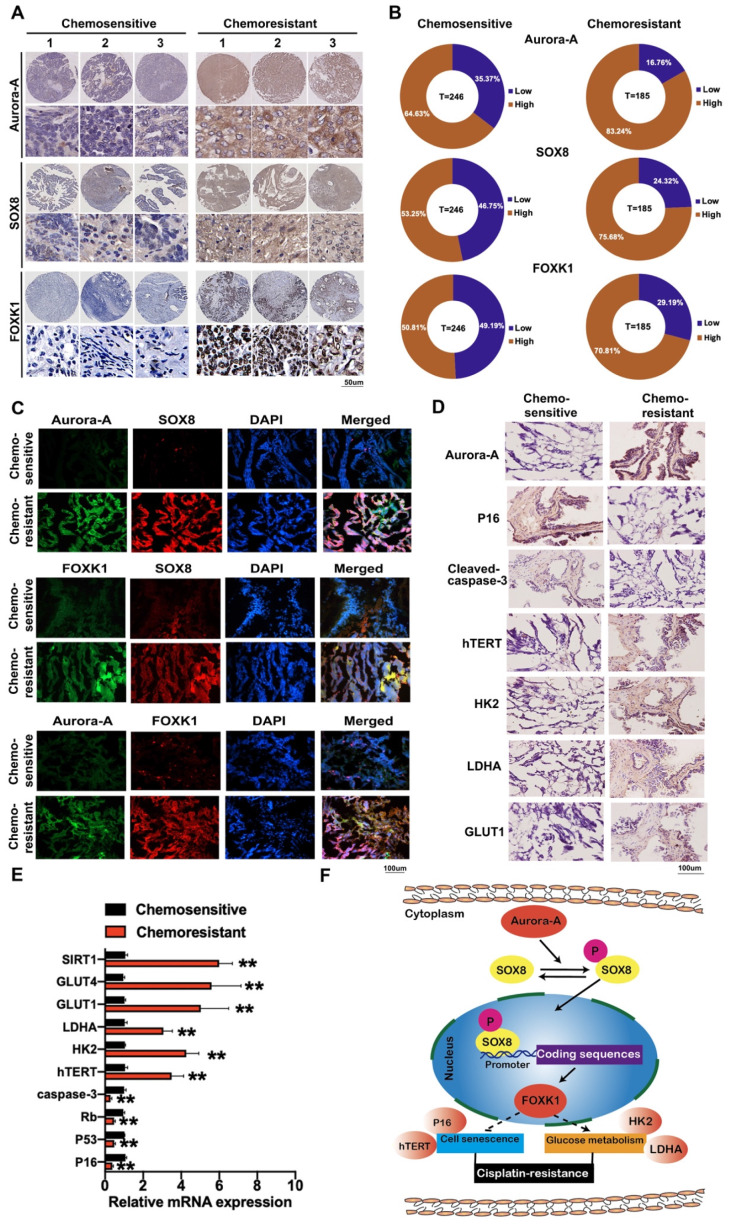
** Immunohistochemistry staining and immunofluorescence of Aurora-A, SOX8, FOXK1 and proteins related to glycolysis and cell senescence. (A)** Representative images of immunohistochemistry staining of Aurora-A, SOX8, and FOXK1 in cisplatin-sensitive and cisplatin-resistant ovarian cancer tissues. **(B)** Relatively high or low expression ratio of Aurora-A, SOX8 and FOXK1 in cisplatin-sensitive and cisplatin-resistant ovarian cancer samples. **(C)** Immunofluorescence staining of Aurora-A, SOX8 and FOXK1.** (D)** Immunohistochemistry of Aurora-A and the proteins associated with cell senescence and glycolysis. **(E)** The mRNA expression levels of genes associated with cell senescence and glycolysis were tested by qRT-PCR assay. **(F)** Schematic model showing the role of the Aurora-A/SOX8/FOXK1 signaling axis in the regulation of cell senescence, glycolysis and chemosensitivity.

To determine the clinical significance of Aurora-A, SOX8, and FOXK1 in ovarian cancer, we assessed their expression patterns using cisplatin-sensitive and -resistant ovarian cancer tissue microarrays (n = 246 and 185, respectively). Aurora-A was expressed at high levels in 83.24% (154/185) chemoresistant and 64.63% (159/246) chemosensitive ovarian cancer issues. Overall, 75.68% (140/185) chemoresistant ovarian cancer tissues showed high expression of SOX8 relative to 53.25% (131/246) chemosensitive ovarian cancer tissues. We observed "high" expression of FOXK1 in 70.81% (131/185) chemoresistant ovarian cancer tissues and 50.81% (125/246) chemosensitive tissues (**Figure 7A-B**). High Aurora-A (log-rank P = 0.0007), SOX8 (log-rank P <0.0001), and FOXK1 (log-rank P = 0.0050) levels were associated with poor OS in chemosensitive ovarian cancer (**Figure S8A**). Similarly, high Aurora-A, SOX8, and FOXK1 expression was correlated with poor OS in chemoresistant ovarian cancer (P = 0.0035, 0.0001, and 0.0003, respectively; **Figure S8B**). Correlations among Aurora-A, SOX8, and FOXK1 were further confirmed via immunofluorescence (**Figure 7C**). Immunohistochemistry, qRT-PCR and immunofluorescence experiments consistently showed that Aurora-A was more highly expressed in chemoresistant tissues and affected expression levels of proteins associated with cell senescence and glycolysis (**Figure 7D-E, Figure S8C-D**). Our data clearly support involvement of the Aurora-A/SOX8/FOXK1 signaling axis in ovarian chemoresistance (**Figure 7F**).

## Discussion

RNA sequencing of PDOs initially showed that chemoresistance of ovarian cancer is associated low cell senescence, high glycolysis and high Aurora-A expression. Moreover, Aurora-A appears to exert a regulatory role through direct binding and phosphorylation of the transcription factor SOX8 to activate the SOX8/FOXK1 signaling axis, highlighting this pathway as a novel potential therapeutic target for chemoresistant ovarian cancer.

Conventional research models have failed to recapitulate the nature and heterogeneity of cancer, thereby hampering scientific and clinical progress. Organoids can efficiently replicate many structural and functional aspects of their *in vivo* counterpart organs and have therefore been employed in the development of numerous novel human cancer models 24. For instance, organoids from endometrial pathologies have been shown to successfully replicate the mutational landscape of tumors, along with patient-specific drug responses, disease-associated traits and cancer-linked mutations 25. Moreover, organoid culture offers several advantages in cancer metabolic research 10. Here, we established both cisplatin-sensitive and -resistant ovarian cancer PDOs and employed RNA sequencing, qRT-PCR and immunofluorescence studies to explore the mechanisms underlying chemoresistance of ovarian cancer. Our results collectively suggest that Aurora-A induces chemoresistance via regulation of cell senescence and glucose metabolism pathways.

Aurora A controls centrosome maturation, timing of mitotic entry, assembly of the bipolar spindle, and chromosome alignment in metaphase 26 and is associated with aggressive tumor phenotypes 20,27,28. The protein is a key regulatory component of the p53 pathway, phosphorylating p53 at Ser215 and 315, leading to abrogation of p53 DNA binding/transactivation activity and promotion of proteolytic degradation after MDM2-mediated ubiquitination 29,30. The p53 protein exerts its effects by regulating the transcription of genes involved in cell cycle arrest, senescence, apoptosis 31, and glycolysis 32,33. However, the specific functions and mechanisms of action of Aurora-A in cell senescence and glucose metabolism remain largely unexplored.

Experiments in the current study demonstrated that Aurora-A strongly reduces cell senescence and increases glucose metabolism, ultimately promoting chemoresistance of ovarian cancer. We showed for the first time that Aurora-A interacts directly with SOX8 and phosphorylates the protein at Ser327 to further regulate the SOX8/FOXK1 axis, which modulates cell senescence and glycolysis, ultimately leading to cisplatin resistance.

In addition, Aurora-A has oncogenic properties in many caners by regulating oncogenic transcription factors. For example, Aurora-A inhibition promoted proteasomal degradation of c-Myc protein through disruption of the c-Myc/Aurora-A complex 34. Our study verified that c-Myc expression was reduced by Aurora-A knockdown in ovarian cancer cells. Furthermore, Aurora-A silencing-mediated low SOX8 transcription was significantly rescued by c-Myc overexpression. Therefore, Aurora-A not only directly phosphorylates SOX8 but also promotes SOX8 transcription indirectly by regulating c-Myc protein.

SOX8 belongs to the Sex-determining region Y-box family of proteins, which are expressed in multiple stem and progenitor cell types and play important roles in the regulation of embryonic development 35. Accumulating evidence supports the importance of SOX genes in drug resistance and EMT. For example, SOX4 is a tumor promoter shown to contribute to drug resistance and progression in cervical cancer and regulate the EMT program in breast cancer 36. While SOX8 is known to regulate cancer stem-like properties and cisplatin-induced EMT in tongue squamous cell carcinoma through effects on the Wnt/β-catenin pathway 37, its functions and mechanisms of action in cancer, particularly ovarian cancer, have rarely been documented. Our results showed that SOX8 targets FOXK1, thereby regulating its transcription, which has significant impacts on senescence, glycolysis and chemoresistance in ovarian cancer.

FOXK1, a member of the FOX family, plays crucial roles in cell proliferation, metastasis, and metabolism 38. Dysregulation of this protein has been reported in various human cancer types, including colorectal cancer 39, esophageal cancer 40, prostate cancer 41 and hepatocellular carcinoma 42. However, the expression patterns and roles of FOXK1 in ovarian cancer have not been established to date. Here, we showed that FOXK1 inhibits cellular senescence and promotes glycolysis and chemoresistance in ovarian cancer through regulating cell senescence- and glycolysis-associated proteins, such as P16, hTERT, HK2 and LDHA. Moreover, high FOXK1 expression was associated with poor survival in ovarian cancer patients.

Several studies suggest that Aurora-A can regulate BRCA1/2 and other DNA repair proteins. For example, Aurora-A suppresses BRCA2-expressing ovarian cancer cells while its silencing restores the level of BRCA2 and increases the number of DNA repair foci of both BRCA2 and Rad51 after γ-irradiation 11. Given that olaparib has been approved as a maintenance drug after platinum-based therapy in recurrent ovarian cancer patients with somatic or germline BRCA mutations 43, combination of Aurora-A-targeted therapies and olaparib may present a promising chemotherapeutic strategy to enhance chemosensitivity to olaparib.

## Conclusion

In summary, the Aurora-A/SOX8/FOXK1 signaling axis promotes chemoresistance via suppression of cell senescence and induction of glucose metabolism in ovarian cancer organoids and cells. Our findings collectively provide evidence of a novel mechanism underlying the chemoresistance-promoting effects of Aurora-A and present potential therapeutic targets to prevent chemoresistance and progression of ovarian cancer.

## Methods

### Patients and tissue samples

Tissues microarrays were collected from patients with ovarian cancer in Xinhua Hospital, Shanghai Jiaotong University School of Medical from 2008 to 2018, after obtaining the subjects' informed consent. The clinical data were shown in **Table S1**. For chemotherapeutic response, 185 (42.92%) patients were resistant to platinum-based chemotherapy and 246 (57.08%) were sensitive to the same treatment. PFS was calculated at the time from the date of surgery to the occurrence of progression, or relapse. OS was measured as the length of time from the initiation of surgery to death from any cause or until the most recent follow-up. PFS less than 6 months was defined as resistant to the last chemotherapy, or else it was defined as sensitivity to the last chemotherapy. Our study was approved by the Ethics Committee of the Xinhua Hospital, and each clinical investigation was conducted according to the principles expressed in the Declaration of Helsinki consent.

### Organoid formation and culture

Cisplatin-sensitive organoids in our study were generated from cisplatin-sensitive ovarian cancer patients' tissues in the first surgery. The cisplatin-resistant PDOs were gained from ovarian cancer tissues of the cisplatin-resistant patients, who underwent reoperation after failure of cisplatin-based chemotherapy. Then the PDOs were cultured as described below and the drug resistance tests were performed with the treatment of cisplatin for 21 days.

For PDOs generation, fresh tumor tissues were immediately transported to laboratory in Advanced DMEM/F12 supplement with 1 % penicillin streptomycin after obtained in the surgery. Tissues were diced into approximately 2-3 mm sections and then digested in 37°C for 1 hrs. Digestion solution was made of Advanced DMEM/F12 containing type IV Collagenase (Sigma-Aldrich; catalog number C9407). The digested sample was filtered through a 70-μm filter (Falcon; catalog number 352350). The cell suspension was then spun at 1000 RPM for 5 min to create a cell pellet. The pellet was washed with red blood for 2-3 times. Cells of solid tumor were then mixed with growth factor reduced Matrigel (Corning; catalog number CB-40230C), and cells concentration was 10,000/50 μl~20,000 cells/50 μl. Once the Matrigel was solidified, 500 μL of general culture medium was added.

Cultures were overlaid with medium containing specific growth factors as the method of Willert 44 with a few modifications. PDOs were kept in a humidified atmosphere of 5% CO2 and 95% air at 37 °C, and medium was changed every 2~3 days.

### Senescence-associated beta-galactosidase activity (SA-β-gal)

The SA-b-gal activity was widely used for cell senescence test 45,46, which was determined using a senescence-associated β-galactosidase assay Kit (Cell Signaling Technology, US) following the manufacturer's instructions. Positive SA-b-gal staining cells were counted and the positive rate was calculated in our study to evaluate the level of cell senescence.

### Cell lines and culture, and establishment of cisplatin-resistant ovarian cancer cell lines

Ovarian cancer cells (OVCA429 and SKOV3) were purchased from American Type Culture Collection (ATCC, US). Ovarian cancer cell lines resistant to cisplatin (OVCA429-CisR and SKOV3-CisR) were successfully established by exposing cancer cells to gradually increasing doses of cisplatin *in vitro*. All cells were grown in RPMI-1640 (Gibco BRL) plus 10% fetal bovine serum (Gibco, Life Technologies), and were maintained in a 5% CO2 incubator at 37 °C.

### Whole-body ^18^F-FDG positron emission tomography/ computed tomography (PET/CT)

The Whole-body ^18^F-FDG positron emission tomography/ computed tomography imaging was performed as previously described 47. Patients fasted for at least 8 h to ensure low levels of serum glucose. Scanning started 1 h after intravenous injection of the tracer (7.4 MBq/kg). The images were acquired on a Siemens biograph 16HR PET/CT scanner with a transaxial intrinsic spatial resolution of 4.1 mm. Quantification of metabolic activity was acquired using the standard uptake value (SUV) normalized to body weight, and the SUVmax for each lesion was calculated.

### Plasmid construction and viral infection

To enhance the expression of Aurora-A, human wide type cDNA of Aurora-A was inserted into pCDH (System Biosciences). Lentiviral vectors were produced and used to infect OVCA429 and SKOV3 cells to generate Aurora-A overexpression cell lines: OVCA429/Aur-OE and SKOV3/Aur-OE. The control cell lines were generated by infection of the same cell lines with viruses containing empty vectors (OVCA429/Vector and SKOV3/Vector). Cell lines with SOX8 overexpression were established with the same methods.

In our study, RNA interference technology was used to generate Aurora-A, SOX8 or FOXK1 knockdown cell lines. The control cell lines were generated by infection of lentiviruses harboring scrambled shRNA expression vector (Con). The specific shRNAs against Aurora-A (Aur-i1/2), SOX8 (SOX8-i1/2) or FOXK1 (FOXK1i) were transduced into ovarian cancer cells. The shRNA sequences of Aurora-A were as follows: 5'-CACATACCAAGAGACCTACAA-3' (sh1); 5'-CCTGTCTTACTGTCATTCGAA-3' (sh2). The shRNA sequences of SOX8 were as follows: 5'-GCGCTCAAAGCCAAGCCGCAT-3' (sh1); 5'-GCCGGCTCACAGGGCGACTAT-3' (sh2). The shRNA sequences of FOXK1 were depicted below: 5'-CCAUCAAGAUCCAGUUCAC- 3' (FOXK1i).

### RNA-sequencing data analysis

RNA-sequencing assay was performed as previously described 47. Briefly, a minimum of 1 mg of total RNA was isolated from ovarian cancer cells or organoids and treated with VAHTS mRNA Capture Beads (Vazyme) to enrich polyAþ RNA before constructing RNA libraries. RNA library preparation was performed by using VAHTS mRNA-seq v2 Library Prep Kit from Illumina (Vazyme). Paired-end sequencing was performed with Illumina HiSeq 3000 at RiboBio Co., Ltd. For computational analysis of RNA-sequencing data, sequencing reads were aligned using the spliced read aligner HISAT2, which was supplied with the Ensemble Human Genome Assembly (Genome Reference Consortium GRCh38) as the reference genome. The gene expression levels for each transcript were estimated as the number of reads per kilobase of exon model per million mapped reads (RPKM). Gene Set Enrichment Analysis (GSEA) was used for gene functional annotation. A gene is considered significantly differentially expressed if its expression differs between any two samples with the fold change > 2 and the p value < 0.05 as calculated by Cufflinks.

### Immunohistochemistry (IHC) assay

The tissue microarray (TMA) was made by the Xinhua hospital. Immunohistochemistry was performed on 7-mm-thick TMA sections using the antibody against Aurora-A (ab13824, Abcam, 1:100 dilution), SOX8 (ab221053, Abcam, 1:100 dilution) and FOXK1 (ab18196, Abcam, 1:100 dilution). Each case was photographed and scored by two independent pathologists. The immunoreactive score (IRS) was multiplicity of the staining intensity and positive cancer percentage. Finally, the assessment of protein expression was defined as negative (≤1+) and positive (>2+ to ≤3+).

### Detection and quantification of the metabolic profile

Metabolites were extracted and analyzed as previously described 48. Briefly, metabolites were extracted from ovarian cancer organoids. Metabolite levels were normalized to the total of all metabolites detected.

### Immunoblotting assay

For biochemical analysis of tissues or cells, lysates were equilibrated for protein levels using a BCA protein assay kit (Pierce, US) and resolved on 8-12% SDS-PAGE gels depending on the experiment. Nuclear and cytoplasmic fractions were isolated using a NE-PER nuclear and cytoplasmic extraction kit (Thermofisher) under manufacturers' conditions. The primary antibodies used in immunoblotting experiments were listed in **Table S2.**

### Quantitative real time PCR (qRT-PCR)

Total RNA was extracted from cells, organoids or tissue samples with Trizol reagent (Invitrogen, US), and it was reverse transcribed using miScript Reverse Transcription Kit (Qiagen, Germany). The primers for mRNA are listed in **Table S3**. The quantification was performed with QuantiTect Probe RT-PCR (Qiagen, Germany). The comparative threshold cycle method was used to determine gene relative expression.

### Immunofluorescence assay (IF)

Cells, organoids or frozen sections of ovarian cancer tissues were fixed with 4% paraformaldehyde for 15 min, and permeabilized with 0.3% Triton X-100 for 15min, followed by blockade with 5% goat serum (Life Technologies, US) for 1 h at room temperature. Then, sections were incubated with primary antibodies overnight at 4°C Secondary antibodies were added, followed by staining with DAPI (Life Technologies, US). Stained sections were imaged using a Leica SP5 confocal fluorescence microscope. The primary antibodies used in IF experiments were listed in **Table S2.**

### Luciferase reporter assay

Luciferase reporter assay was performed as previously described 49. Cells were transfected with the CRE-luciferase (pGL3 vector), and luciferase activity was measured in the GloMax-Multi Microplate Reader (Promega). Renilla luciferase was used to normalize reporter luciferase activities.

### Chromatin immunoprecipitation (ChIP) assay

A Pierce Agarose ChIP Kit (Thermo, #27177) was used for ChIP assay following the manufacturer's guidance. Chromatin was mechanically sheared using sonication after SKOV3-CisR cells were collected and crosslinked by formaldehyde and then subjected to immunoprecipitation with 4 mL IgG (Cell Signaling Technology), 10 mL SOX8 (ab 207082, Abcam), or 2 mL Polymerase II (Imgenex) antibodies. Immuno-precipitated and total chromatin was then reverse cross-linked and recovered using column purification. The amount of DNA was further assessed by qRT-PCR, using the FOXK1-specific ChIP primers and SYBR Select Master Mix (Applied Biosystems, Grand Island, NY, USA).

### Immunoprecipitation (IP)

Immunoprecipitation was performed using Pierce Crosslink Immunoprecipitation Kit (Thermo, #26147) as per manufacturer's protocol. Briefly, cells were lysed in RIPA lysis buffer (25mM Tris-HCl, pH 7.4, 0.15M NaCl, 0.001M EDTA, 1% NP-40, 5% glycerol) in the presence of a protease inhibitor cocktail mixture (Sigma-Aldrich). Cells lysates were pre-cleared by the control agarose resin and then immunoprecipitated using anti-Aurora-A antibody (ab13824, Abcam) overnight at 4°C. Antigen was eluted and subjected to SDS page electrophoresis.

### Mass Spectrometry (MS)

The Aurora-A-SOX8 complex was obtained by IP with anti-Aurora-A from SKOV3-CisR cells according to the method above. The eluted proteins were resolved on gradient SDS-PAGE, silver stained, and subjected to MS sequencing and data analysis. In-solution and in-gel digestion were performed according to a previously published method 50. Briefly, gel bands were minced and destained with 50% acetonitrile in 50 mM ammonium bicarbonate. Proteins were reduced with 10 mM DTT at 56℃, followed by alkylation with 55 mM iodoacetamide at room temperature in the dark. Trypsin digestion was performed overnight at 37℃ with gentle shaking. Peptides were extracted using 1% trifluoroacetic acid in 50% acetonitrile. Samples were vacuum-dried and reconstituted in 0.1% formic acid for subsequent MS analysis. The treated samples were examined by nanoLC-MS/MS (nanoACQUITY UPLC and SYNAPT G2 HD mass spectrometer, Waters). MS/MS data were obtained with Data Dependent Analysis mode and processed with PLGS 2.4 software (Waters), and the resulting peak list was searched against the NCBI database with the MASCOT search engine. All the Aurora-A-binding proteins tested by MS after IP were listed in **Table S4.**

### Fӧrster resonance energy transfer-fluorescence lifetime imaging (FRET-FLIM)

We performed FRET-FLIM experiments as previously described 47. Briefly, acceptor proteins (fused to RFP) and donor proteins (fused to GFP) were expressed from vector CMV3-C-OFPSpark and pCMV3-C-GFPSpark, respectively. The FRET-FLIM experiments were performed on a Leica TCS SMD FLCS confocal microscope excitation with WLL (white light laser) and emission collected by an SMD SPAD (single photon-sensitive avalanche photodiodes) detector.

### *In vitro* kinase assay

Construct for GST-tagged SOX8 was transformed to *Escherichia coli* and induced with 0.1 mM IPTG (Sigma- Aldrich) overnight at 16 °C and then purified using glutathione-Sepharose 4B beads (GE Healthcare). The in vitro kinase assay for wild-type or mutant Aurora-A was carried out as previously described 51,52. The immunoprecipitated Aurora-A was incubated for 15 min with the recombinant GST-SOX8 in phosphorylation buffer supplemented with 2.5 protease and phosphatase inhibitor cocktail (P1045, Beyotime). After denaturation by adding 5×SDS/PAGE sample loading buffer and boiling at 100°C for 5 min, the samples were analyzed by western blotting.

### Glycolysis analysis

The glycolysis process was tested by the Lactate Colorimetric Assay Kit (BioVision), Glucose Uptake Colorimetric Assay Kit (BioVision), Amplite Colorimetric NADPH Assay Kit (AAT Bioquest Inc.) and ATP Assay Kit (SIGMA ALOR- ICH) following the manufacturer's protocols.

### Oxygen consumption rate (OCR) and extracellular acidification rate (ECAR)

Cellular mitochondrial function was measured using the Seahorse XF Cell Mito stress test Kit and the Bioscience XF96 Extracellular Flux Analyzer, according to the manufacturers' instructions. Glycolytic capacity was determined using the Glycolysis Stress Test Kit as per the manufacturer's instructions. Briefly, 4
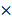
10^4^ cells were seeded onto 96-well plates and incubated overnight. After washing the cells with Seahorse buffer, 175 mL of Seahorse buffer plus 25 mL each of 1 mmol/L oligomycin, 1 mmol/L FCCP, and 1 mmol/L rotenone was automatically injected to measure the OCR. Then, 25 mL each of 10 mmol/L glucose, 1 mmol/L oligomycin, and 100 mmol/L 2-deoxy-glucose were added to measure the ECAR. The OCR and ECAR values were calculated after normalization to the cell number and were plotted as the mean SD.

### Cell counting kit-8 (CCK-8) cell viability assay

The inhibition rate of cell viability was tested by CCK-8. We plated 5 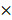 10^3^ cancer cells per well in 96-well plates. The next day, cells were treated with various concentrations of cisplatin (2.5, 5, 10, 20, 40, 80, 160 ug/mL). After 48h the cell viability was tested by CCK-8 (Dojindo Laboratories) according to the manufacturer's instructions. Cells were incubated with 10 μl of CCK-8 diluted in normal culture medium at 37°C for 2 h every day, and then OD measurements at 450 nm were tested.

The half-maximal inhibitory concentration (IC50) was calculated by the algebraic formula of the improved Karber method (lgIC50 = Xm-I[P-(3-Pm-Pn)/4]; Xm: lg (maximum dose), I: lg (maximum dose / adjacent dose), P: sum of the positive reaction rates, Pm: maximum positive reaction rate, Pn: minimum positive reaction rate). The experiment was repeated three times in triplicate.

### Subcellular fractionation

Nuclear and cytosolic fractionation of ovarian cancer cells were obtained by the nuclear and cytoplasmic extraction Kit (Tiangen Biotech) according to the manufacturer's instructions.

### Flow cytometry

To determine the levels of apoptosis, cells were treated with 5ug/mL cisplatin for 48h and then collected using trypsin, washed twice with chilled 1xPBS and resuspended in 200 mL binding buffer at a density of 1 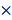 10^7^ cells/mL. Next, cells were stained with 5 mL Annexin V and propidium iodide using an apoptosis detection kit (556547, BD Biosciences) and subjected to analysis by flow cytometry (Cytomics FC 500 MPL, Beckman Coulter). Early apoptosis was determined based on the percentage of cells with Annexin V positive, while late apoptotic cells were defined as double positive for PI and Annexin V. Experiments were done at least three times for final analyses.

### Mouse studies

Experimental procedures and animal care in our study were performed following Guidelines for Animal Experiments and were approved by the Ethics Committee at Xinhua hospital. Four-week-old female BALB/c nude mice (Department of Laboratory Animals, Xinhua hospital) were subcutaneously injected with Aurora-A high expression SKOV3-CisR cells and Aurora-A knockdown SKOV3-CisR cells (5 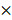 106 cells for each mouse, respectively). Tumor volumes were calculated with the following formula: *V (volume) = L (length)* 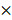
* W (width)^2^* 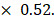 Once reaching an average tumor volume of 100 mm^3^, part of the mice were intraperitoneally treated with cisplatin (3 mg/kg). The glucose uptake of the tumor was evaluated by SUVmax of PET/CT scan. Measurement of tumor volume was done every 3 days. Finally, mice were sacrificed and all the tumors were dissected and weighed.

### Statistical analysis

The data in this study were calculated using GraphPad Prism and reported as mean ± SD. Comparisons between controls and treated groups were determined by paired t test or one-way ANOVA followed by Tukey multiple comparison tests. Clinico-pathologic characteristics analysis was performed using SPSS software (SPSS Standard Version 24.0, SPSS Inc. Chicago, IL). The Kaplan-Meier method with log-rank analyses were used to obtain estimates of OS. Variables with a value of P < 0.05 in univariate analysis were included in the subsequent multivariate analysis on the basis of the Cox proportional hazards model. A probability less than 0.05 was considered statistically significantly different.

## Supplementary Material

Supplementary figures and tables.

## Abbreviations

Aur-A: Aurora-A
SOX8: sex determining region Y-box 8
FOXK1: forkhead-box k1
PFS: progression-free survival
OS: overall survival
TME: tumor microenvironment
PDOs: patient derived organoids
SA-β-gal or SABG: senescence-associated beta-galactosidase
STR: short tandem repeat
PET/CT: positron emission tomography/computed tomography
SUV: standard uptake value
FPKM: fragments per kilobase of transcript per million mapped reads
GSEA: gene set enrichment analysis
TMA: tissue microarray
IRS: immunoreactive score
RT-qPCR: reverse transcription quantitative real-time polymerase chain reaction
IF: immunofluorescence assay
ChIP: chromatin immunoprecipitation
IP: immunoprecipitation
MS: mass spectrometry
FRET: fӧrster resonance energy transfer
FLIM: fluorescence lifetime imaging
WLL: white light laser
OCR: oxygen consumption rate
ECAR: extracellular acidification rate
CCK-8: cell counting kit-8

## References

[1] Siegel RL, Miller KD, Jemal A (2018). Cancer statistics, 2018. CA Cancer J Clin.

[2] Wang Z, Liu H, Xu C (2018). Cellular senescence in the treatment of ovarian cancer. Int J Gynecol Cancer.

[3] Berrino F, De Angelis R, Sant M, Rosso S, Bielska-Lasota M, Coebergh JW (2007). Survival for eight major cancers and all cancers combined for European adults diagnosed in 1995-99: results of the EUROCARE-4 study. Lancet Oncol.

[4] Mukherjee A, Huynh V, Gaines K, Reh WA, Vasquez KM (2019). Targeting the high-mobility group box 3 protein sensitizes chemoresistant ovarian cancer cells to cisplatin. Cancer Res.

[5] Davis A, Tinker AV, Friedlander M (2014). "Platinum resistant" ovarian cancer: what is it, who to treat and how to measure benefit?. Gynecol Oncol.

[6] Kopper O, de Witte CJ, Lohmussaar K, Valle-Inclan JE, Hami N, Kester L (2019). An organoid platform for ovarian cancer captures intra- and interpatient heterogeneity. Nat Med.

[7] van de Wetering M, Francies HE, Francis JM, Bounova G, Iorio F, Pronk A (2015). Prospective derivation of a living organoid biobank of colorectal cancer patients. Cell.

[8] Vlachogiannis G, Hedayat S, Vatsiou A, Jamin Y, Fernandez-Mateos J, Khan K (2018). Patient-derived organoids model treatment response of metastatic gastrointestinal cancers. Science.

[9] Sachs N, de Ligt J, Kopper O, Gogola E, Bounova G, Weeber F (2018). A living biobank of breast cancer organoids captures disease heterogeneity. Cell.

[10] Drost J, Clevers H (2018). Organoids in cancer research. Nat Rev Cancer.

[11] Sun H, Wang Y, Wang Z, Meng J, Qi Z, Yang G (2014). Aurora-A controls cancer cell radio- and chemoresistance via ATM/Chk2-mediated DNA repair networks. Biochim Biophys Acta.

[12] Yin T, Zhao ZB, Guo J, Wang T, Yang JB, Wang C (2019). Aurora A inhibition eliminates myeloid cell-mediated immunosuppression and enhances the efficacy of anti-PD-L1 therapy in breast cancer. Cancer Res.

[13] Wang LH, Xiang J, Yan M, Zhang Y, Zhao Y, Yue CF (2010). The mitotic kinase Aurora-A induces mammary cell migration and breast cancer metastasis by activating the Cofilin-F-actin pathway. Cancer Res.

[14] Chen J, Li D, Wei C, Sen S, Killary AM, Amos CI (2007). Aurora-A and p16 polymorphisms contribute to an earlier age at diagnosis of pancreatic cancer in Caucasians. Clin Cancer Res.

[15] Cammareri P, Scopelliti A, Todaro M, Eterno V, Francescangeli F, Moyer MP (2010). Aurora-a is essential for the tumorigenic capacity and chemoresistance of colorectal cancer stem cells. Cancer Res.

[16] Lassmann S, Shen Y, Jutting U, Wiehle P, Walch A, Gitsch G (2007). Predictive value of Aurora-A/STK15 expression for late stage epithelial ovarian cancer patients treated by adjuvant chemotherapy. Clin Cancer Res.

[17] Chiba Y, Sato S, Itamochi H, Yoshino N, Fukagawa D, Kawamura H (2017). Inhibition of Aurora kinase A synergistically enhances cytotoxicity in ovarian clear cell carcinoma cell lines induced by cisplatin: a potential treatment strategy. Int J Gynecol Cancer.

[18] Scharer CD, Laycock N, Osunkoya AO, Logani S, McDonald JF, Benigno BB (2008). Aurora kinase inhibitors synergize with paclitaxel to induce apoptosis in ovarian cancer cells. J Transl Med.

[19] VanderPorten EC, Taverna P, Hogan JN, Ballinger MD, Flanagan WM, Fucini RV (2009). The Aurora kinase inhibitor SNS-314 shows broad therapeutic potential with chemotherapeutics and synergy with microtubule-targeted agents in a colon carcinoma model. Mol Cancer Ther.

[20] Nikonova AS, Astsaturov I, Serebriiskii IG, Dunbrack RL Jr, Golemis EA (2013). Aurora A kinase (AURKA) in normal and pathological cell division. Cell Mol Life Sci.

[21] Brunner AM, Blonquist TM, DeAngelo DJ, McMasters M, Fell G, Hermance NM (2020). Alisertib plus induction chemotherapy in previously untreated patients with high-risk, acute myeloid leukaemia: a single-arm, phase 2 trial. Lancet Haematol.

[22] Park SI, Lin CP, Ren N, Angus SP, Dittmer DP, Foote M (2019). Inhibition of Aurora A kinase in combination with chemotherapy induces synthetic lethality and overcomes chemoresistance in Myc-overexpressing lymphoma. Target Oncol.

[23] Yang N, Wang C, Wang J, Wang Z, Huang D, Yan M (2019). Aurora kinase A stabilizes FOXM1 to enhance paclitaxel resistance in triple-negative breast cancer. J Cell Mol Med.

[24] Tuveson D, Clevers H (2019). Cancer modeling meets human organoid technology. Science.

[25] Boretto M, Maenhoudt N, Luo X, Hennes A, Boeckx B, Bui B (2019). Patient-derived organoids from endometrial disease capture clinical heterogeneity and are amenable to drug screening. Nat Cell Biol.

[26] Shagisultanova E, Dunbrack RL Jr, Golemis EA (2015). Issues in interpreting the in vivo activity of Aurora-A. Expert Opin Ther Targets.

[27] Katayama H, Sen S (2011). Functional significance of Aurora kinase A regulatory interactions with p53-ERalpha complex in human breast cancer cells. Horm Cancer.

[28] Korobeynikov V, Borakove M, Feng Y, Wuest WM, Koval AB, Nikonova AS (2019). Combined inhibition of Aurora A and p21-activated kinase 1 as a new treatment strategy in breast cancer. Breast Cancer Res Treat.

[29] Katayama H, Sasai K, Kawai H, Yuan ZM, Bondaruk J, Suzuki F (2004). Phosphorylation by aurora kinase A induces Mdm2-mediated destabilization and inhibition of p53. Nat Genet.

[30] Liu Q, Kaneko S, Yang L, Feldman RI, Nicosia SV, Chen J (2004). Aurora-A abrogation of p53 DNA binding and transactivation activity by phosphorylation of serine 215. J Biol Chem.

[31] Riley T, Sontag E, Chen P, Levine A (2008). Transcriptional control of human p53-regulated genes. Nat Rev Mol Cell Biol.

[32] Zhou Y, Niu W, Luo Y, Li H, Xie Y, Wang H (2019). p53/Lactate dehydrogenase A axis negatively regulates aerobic glycolysis and tumor progression in breast cancer expressing wild-type p53. Cancer Sci.

[33] Kruiswijk F, Labuschagne CF, Vousden KH (2015). p53 in survival, death and metabolic health: a lifeguard with a licence to kill. Nat Rev Mol Cell Biol.

[34] Li Y, Li X, Pu J, Yang Q, Guan H, Ji M (2018). c-Myc is a major determinant for antitumor activity of Aurora A kinase inhibitor MLN8237 in thyroid Cancer. Thyroid.

[35] Sarkar A, Hochedlinger K (2013). The sox family of transcription factors: versatile regulators of stem and progenitor cell fate. Cell Stem Cell.

[36] Sun R, Jiang B, Qi H, Zhang X, Yang J, Duan J (2015). SOX4 contributes to the progression of cervical cancer and the resistance to the chemotherapeutic drug through ABCG2. Cell Death Dis.

[37] Xie SL, Fan S, Zhang SY, Chen WX, Li QX, Pan GK (2018). SOX8 regulates cancer stem-like properties and cisplatin-induced EMT in tongue squamous cell carcinoma by acting on the Wnt/beta-catenin pathway. Int J Cancer.

[38] Shi X, Wallis AM, Gerard RD, Voelker KA, Grange RW, DePinho RA (2012). Foxk1 promotes cell proliferation and represses myogenic differentiation by regulating Foxo4 and Mef2. J Cell Sci.

[39] Wu M, Wang J, Tang W, Zhan X, Li Y, Peng Y (2016). FOXK1 interaction with FHL2 promotes proliferation, invasion and metastasis in colorectal cancer. Oncogenesis.

[40] Chen D, Wang K, Li X, Jiang M, Ni L, Xu B (2017). FOXK1 plays an oncogenic role in the development of esophageal cancer. Biochem Biophys Res Commun.

[41] Chen F, Xiong W, Dou K, Ran Q (2017). Knockdown of FOXK1 suppresses proliferation, migration, and invasion in prostate cancer cells. Oncol Res.

[42] Li P, Yu Z, He L, Zhou D, Xie S, Hou H (2017). Knockdown of FOXK1 inhibited the proliferation, migration and invasion in hepatocellular carcinoma cells. Biomed Pharmacother.

[43] Pignata S, S CC, Du Bois A, Harter P, Heitz F (2017). Treatment of recurrent ovarian cancer. Ann Oncol.

[44] Willert K, Brown JD, Danenberg E, Duncan AW, Weissman IL, Reya T (2003). Wnt proteins are lipid-modified and can act as stem cell growth factors. Nature.

[45] Lewinska A, Adamczyk-Grochala J, Deregowska A, Wnuk M (2017). Sulforaphane-Induced Cell Cycle Arrest and Senescence are accompanied by DNA Hypomethylation and Changes in microRNA Profile in Breast Cancer Cells. Theranostics.

[46] Yu Y, Qi J, Xiong J, Jiang L, Cui D, He J (2019). Epigenetic Co-Deregulation of EZH2/TET1 is a Senescence-Countering, Actionable Vulnerability in Triple-Negative Breast Cancer. Theranostics.

[47] Cao D, Qi Z, Pang Y, Li H, Xie H, Wu J (2019). Retinoic acid-related orphan receptor C regulates proliferation, glycolysis, and chemoresistance via the PD-L1/ITGB6/STAT3 signaling axis in bladder cancer. Cancer Res.

[48] Finley LW, Carracedo A, Lee J, Souza A, Egia A, Zhang J (2011). SIRT3 opposes reprogramming of cancer cell metabolism through HIF1alpha destabilization. Cancer Cell.

[49] Gan L, Meng J, Xu M, Liu M, Qi Y, Tan C (2018). Extracellular matrix protein 1 promotes cell metastasis and glucose metabolism by inducing integrin beta4/FAK/SOX2/HIF-1alpha signaling pathway in gastric cancer. Oncogene.

[50] Jin BF, He K, Wang HX, Wang J, Zhou T, Lan Y (2003). Proteomic analysis of ubiquitin-proteasome effects: insight into the function of eukaryotic initiation factor 5A. Oncogene.

[51] Yannay-Cohen N, Carmi-Levy I, Kay G, Yang CM, Han JM, Kemeny DM (2009). LysRS serves as a key signaling molecule in the immune response by regulating gene expression. Mol Cell.

[52] Luo C, Yu M, Li S, Huang X, Qi H, Gao X (2020). Methionine stimulates GlyRS phosphorylation via the GPR87-CDC42/Rac1-MAP3K10 signaling pathway. Biochem Biophys Res Commun.

