# Supplementary material for: Aurora-A/SOX8/FOXK1 signaling axis promotes chemoresistance via suppression of cell senescence and induction of glucose metabolism in ovarian cancer organoids and cells
Source: Theranostics. 2020 May 25;10(15):6928–45. doi: 10.7150/thno.43811 (PMC7295065; doi:10.7150/thno.43811)
Supplement: Supplementary file 1 — Supplementary figures and tables. [file thnov10p6928s1.pdf]

Supplemental Figure 1

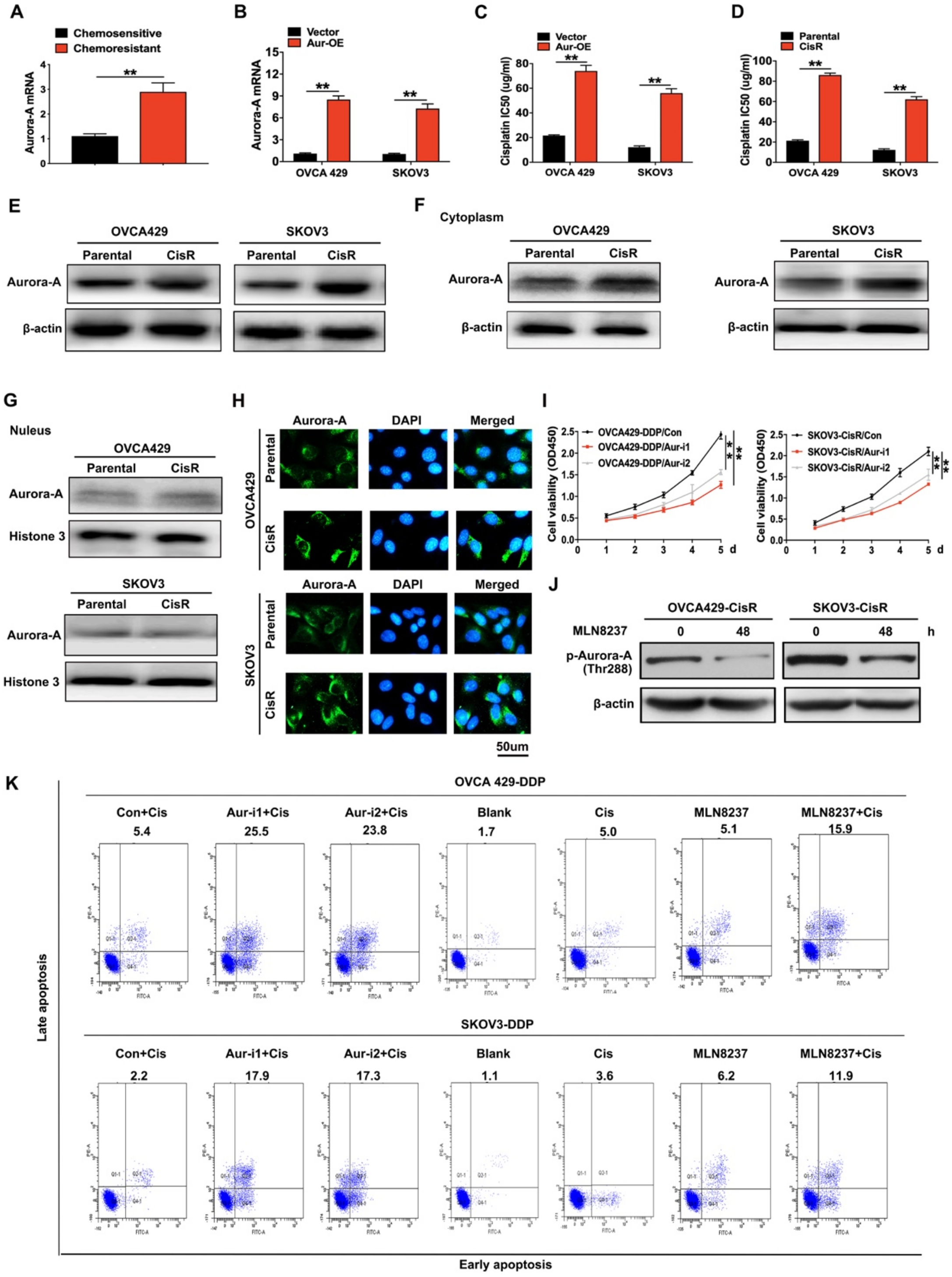

Supplemental Figure 2

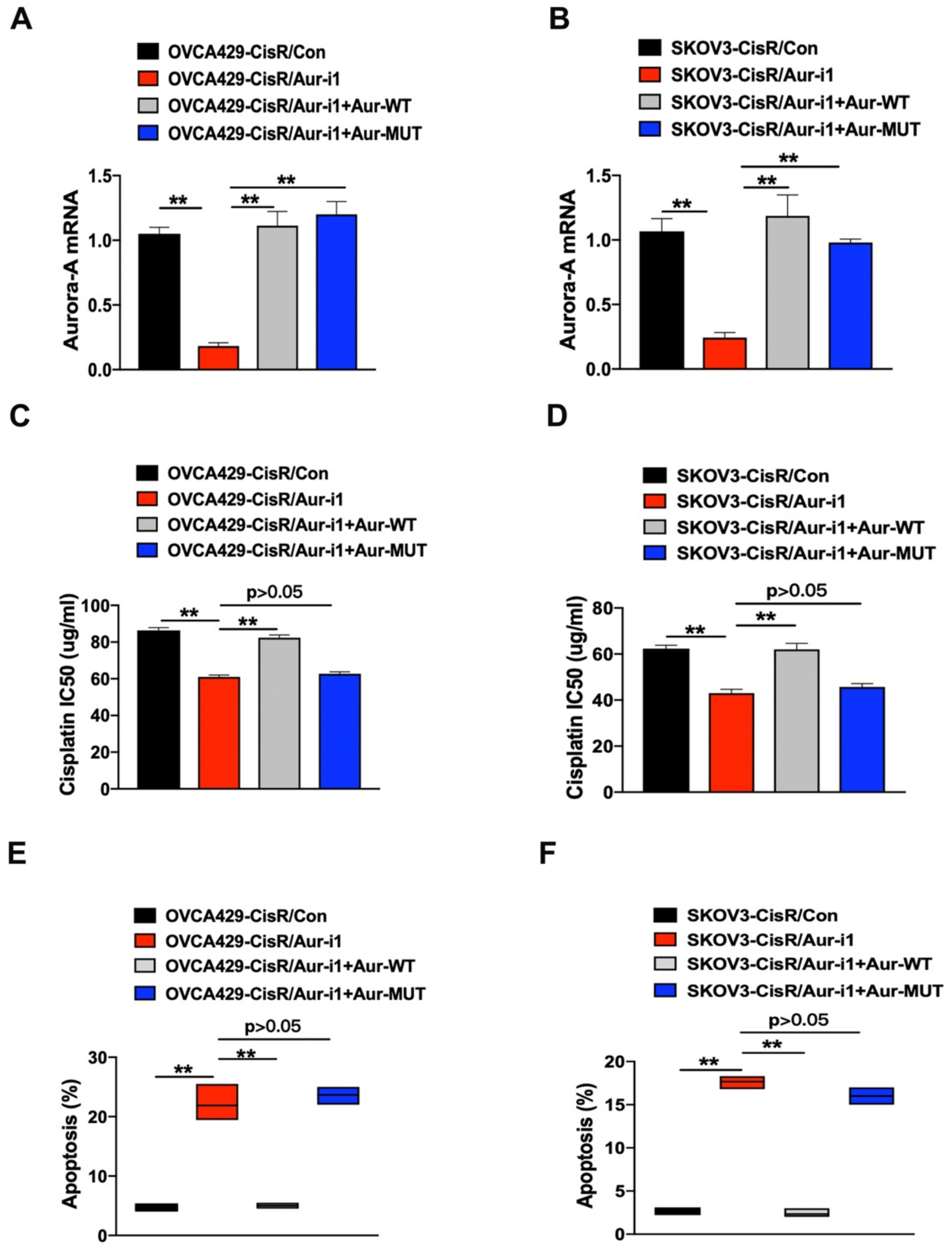

Supplemental Figure 3

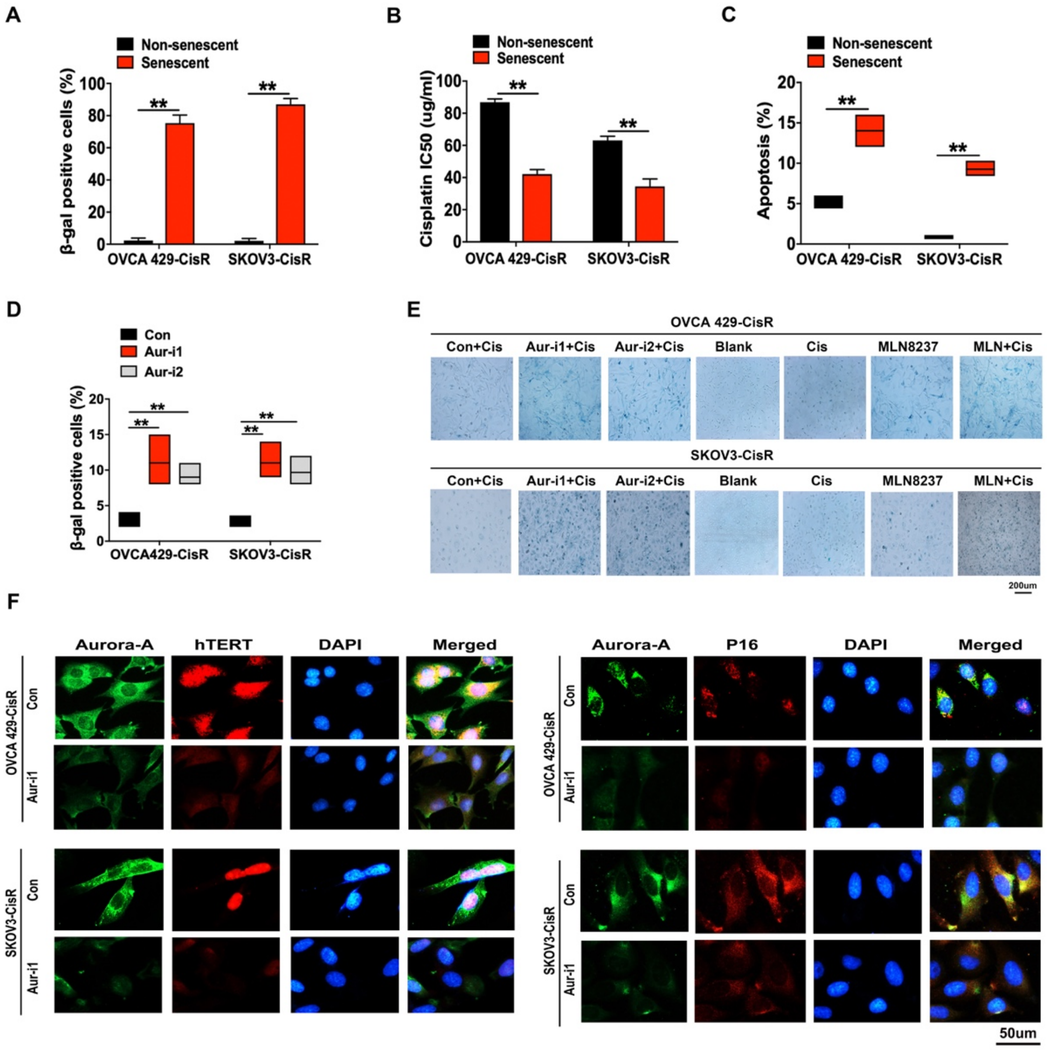

Supplemental Figure 4

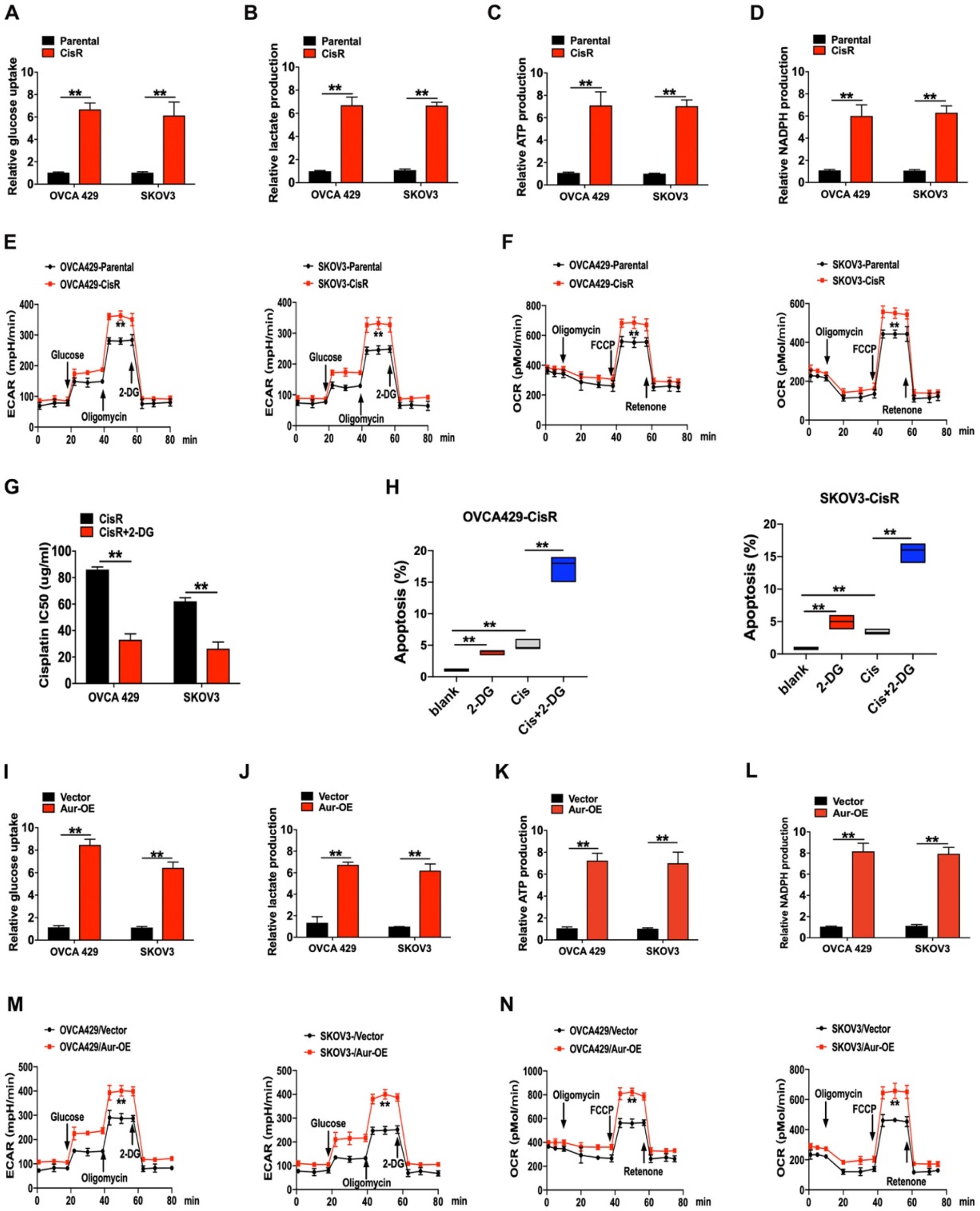

Supplemental Figure 5

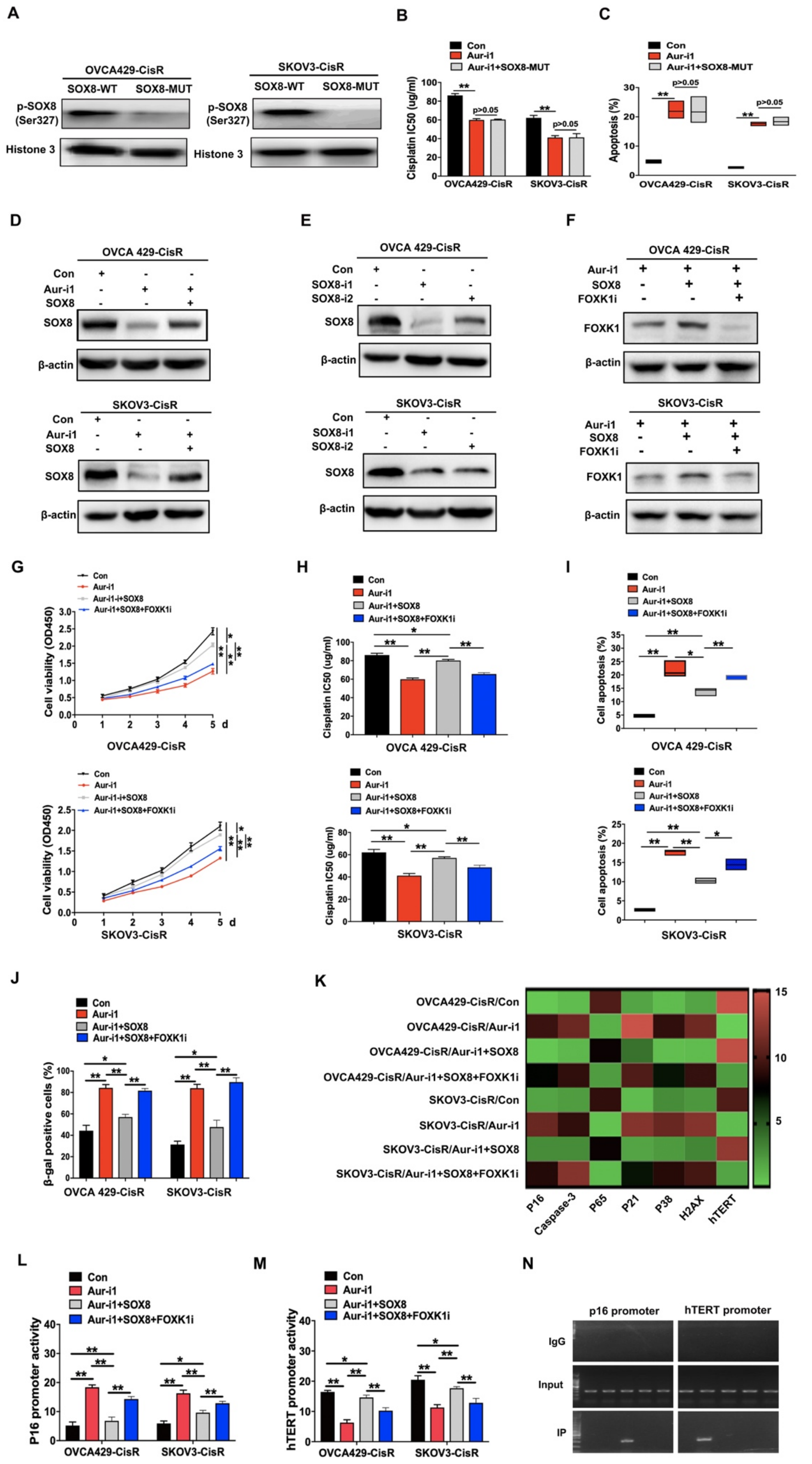

Supplemental Figure 6

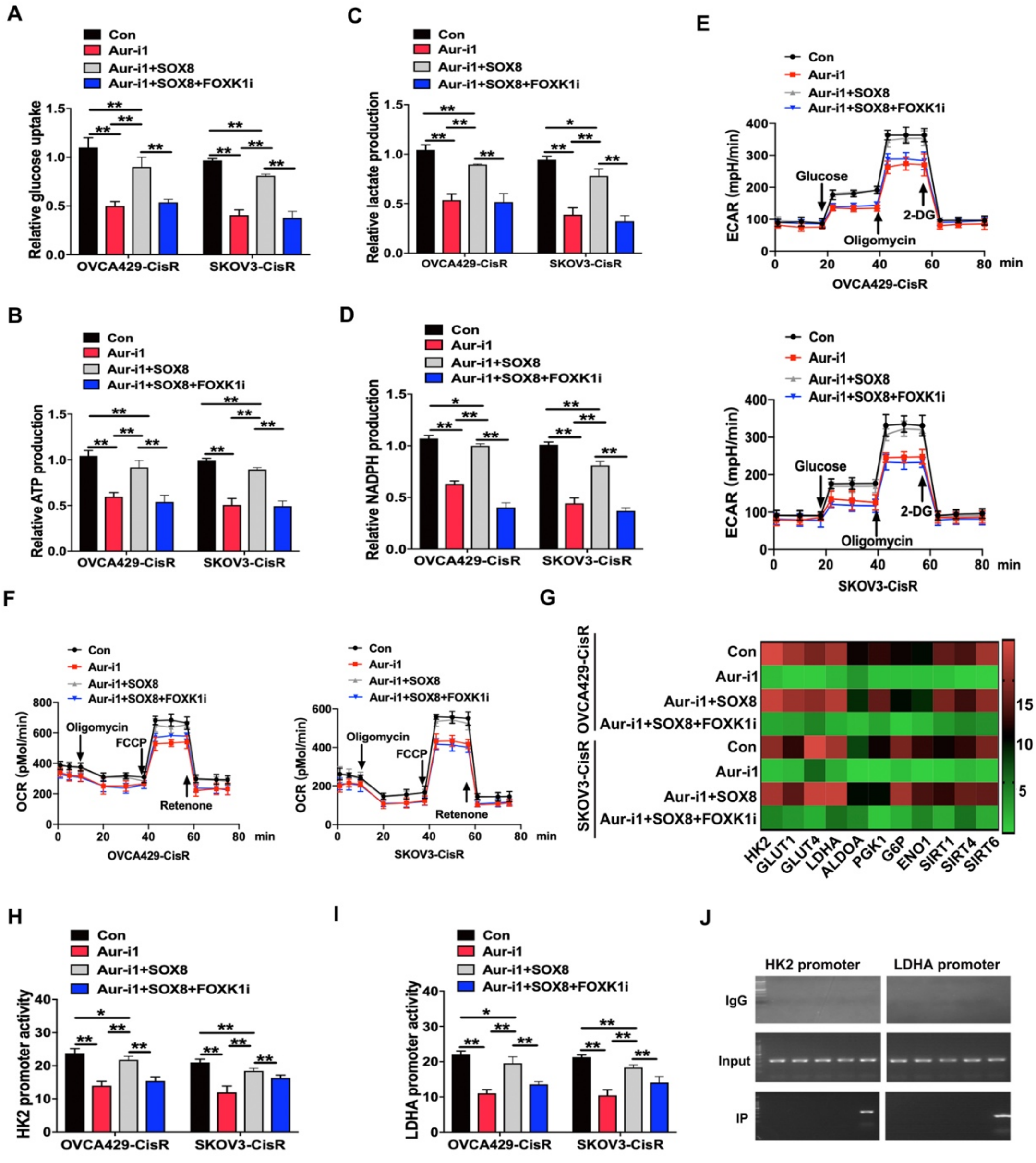

Supplemental Figure 7

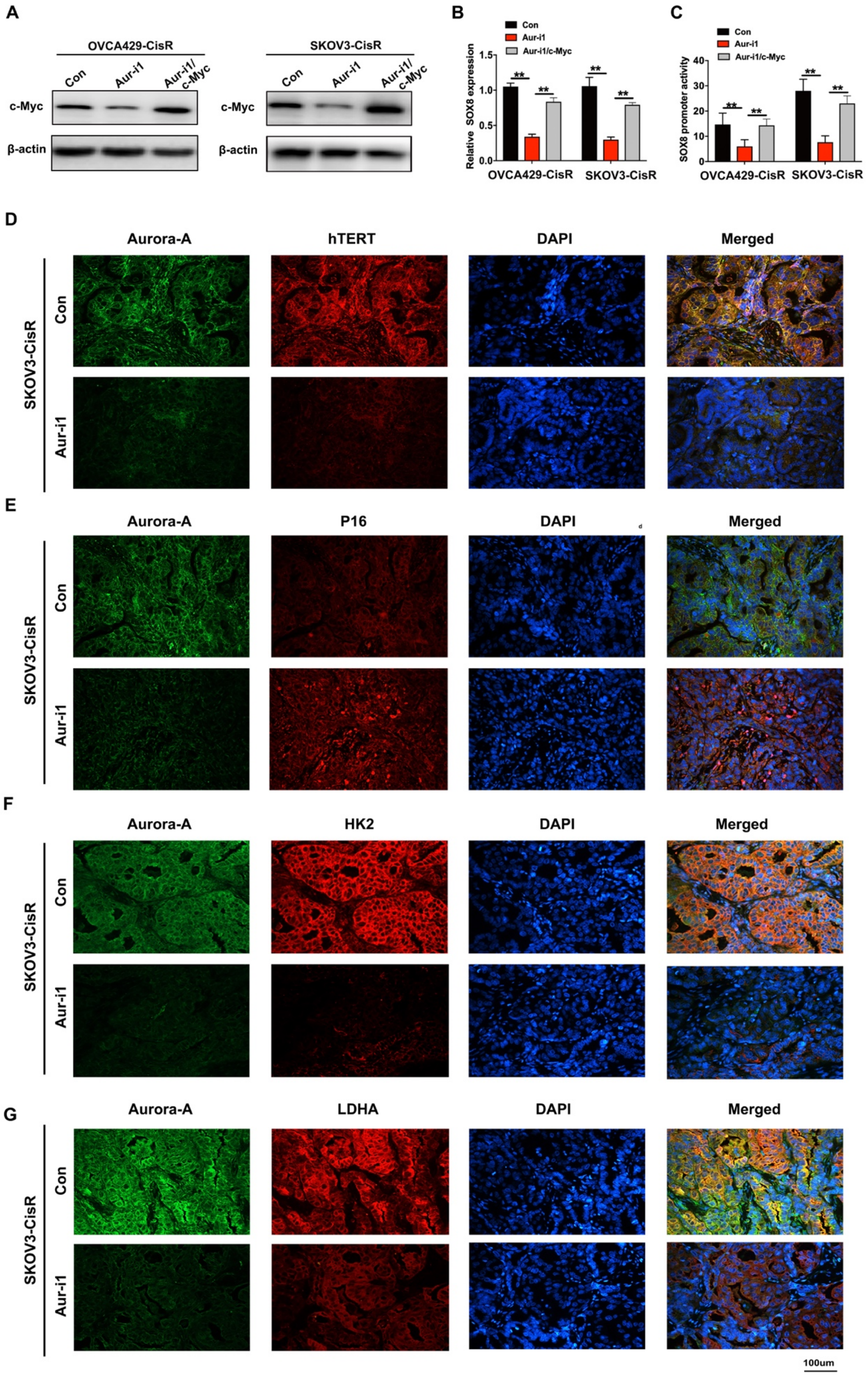

Supplemental Figure 8

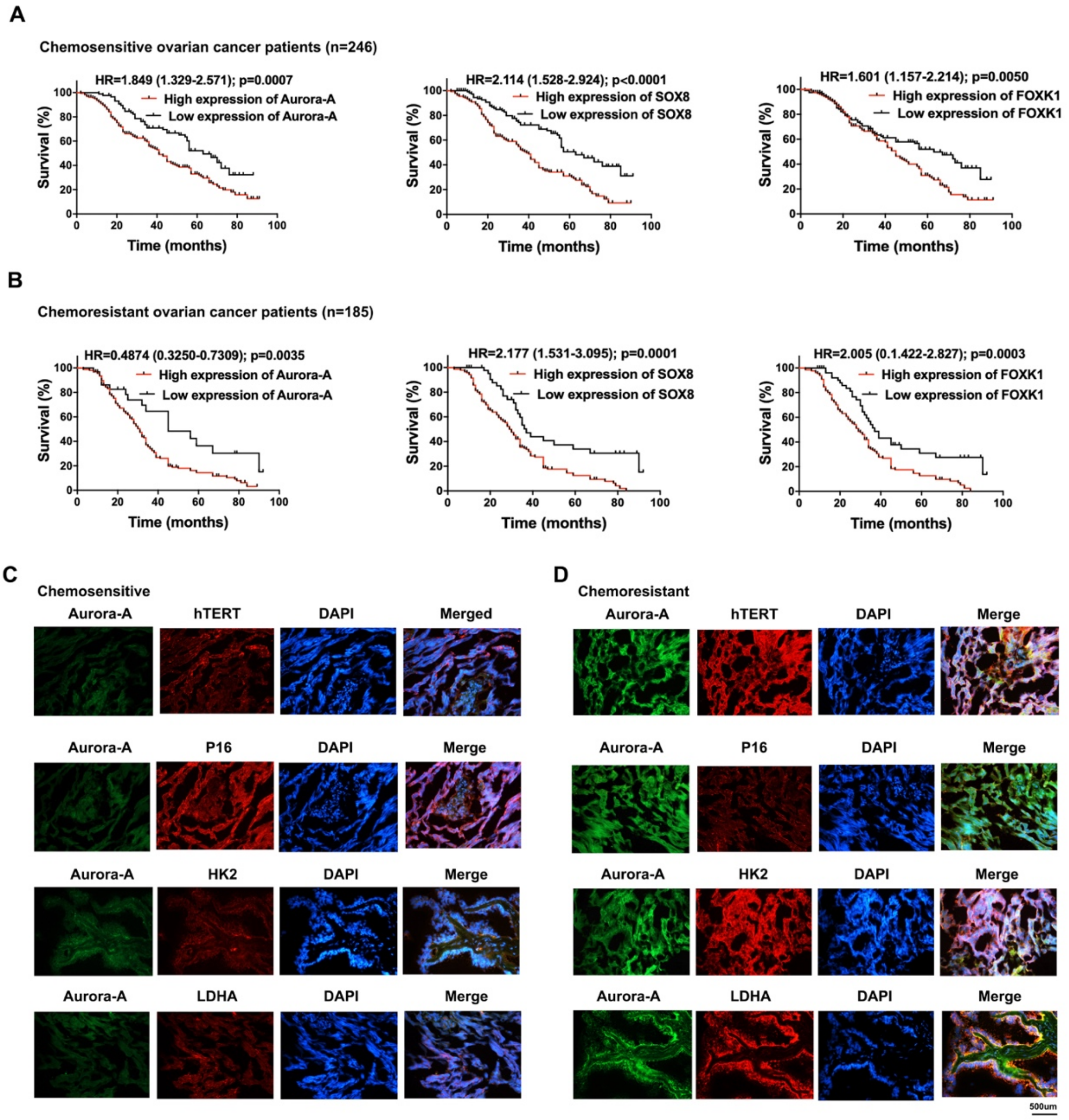

Supplementary figure legends

Figure S1. Establishment of chemoresistant ovarian cancer cell lines and the function of Aurora-A in cell chemoresistance.

(A) qRT-PCR assay detected the relation between Aurora-A and chemoresistance in

cisplatin-sensitive and -resistant ovarian cancer tissues. 40 pairs of tissues were used in the experiment. **, $P < 0.01$.

(B) Aurora-A overexpression was verified by qRT-PCR assay.

(C) and (D) IC50 of cisplatin. Aurora-A overexpression or cisplatin-resistance increased the IC50 of cisplatin in ovarian cancer cell lines.

(E) Aurora-A expression was enhanced in cisplatin-resistant ovarian cancer cells compared with the parental cells.

(F) and (G) Aurora-A distribution in cytoplasm and nucleus of cisplatin-resistant ovarian cancer cells and the parental cells.

(H) Representative images of immunofluorescence assay of Aurora-A.

(I) Cell viability of Aurora-A knockdown ovarian cancer cells and the controls tested by CCK-8 assay. **, $P < 0.01$.

(J) Aurora-A activity was tested by p-Aurora-A (Thr288), and the effect of Aurora-A inhibitor MLN8237（0.5nM for 48h）on p-Aurora-A (Thr288) was verified by immunoblotting.

(K) Representative images of apoptosis measured by flow cytometry in cells stained with annexin V and propidium iodide.

Figure S2. The effect of wild-type or mutant Aurora-A on chemoresistance of ovarian cancer cells.

(A) and (B) Aurora-A mRNA expression was verified by qRT-PCR assay.

(C) and (D) IC50 of cisplatin. Effect of wild-type or mutant Aurora-A on IC50

of cisplatin in ovarian cancer cell lines.

(E) and (F) Apoptosis of ovarian cancer cells induced by wild-type or mutant Aurora-A. Cells were treated with 5ug/mL cisplatin for 48h.

Figure S3. The association between Aurora-A and cell senescence in ovarian cancer cells.

(A) Senescent cells were generated by low dose cisplatin treatment (2ug/ml for 6 days) and verified by positive β-galactosidase staining.

(B) Senescent ovarian cancer cells decreased the IC50 of cisplatin compared with the non-senescent group.

(C) Senescent ovarian cancer cells increased the apoptosis after cisplatin treatment (5ug/mL cisplatin for 48h) compared with the non-senescent group.

(D) Aurora-A knockdown increased the cell senescence tested by positive β-galactosidase staining. Cells were expanded for 15 passages.

(E) Representative images of β-galactosidase staining in ovarian cancer cells. Cells were treated with the same method as Figure 2F-G**.**

(F) Representative images of immunofluorescence assay of Aurora-A and proteins associated with cell senescence.

Figure S4 The association between glycolysis and chemoresistance, and the effect of Aurora-A overexpression on glycolysis.

(A), (B), (C) and (D) Glucose uptake (**A**), lactate (**B**), ATP (**C**) and NADPH (**D**) production were determined as described in Methods. Parental cells were the relative chemosensitive cells compared with the cisplatin-resistant group.

(E) and (F) ECAR (**E**) and OCR (**F**) were determined as described in Methods.

(G) and (H) Glycolysis inhibitor 2-DG (5mM for 48h) decreased the IC50 of cisplatin, and increased the apoptosis induced by cisplatin (5ug/mL for 48h) in ovarian cancer cells.

(I), (J), (K) and (L) Glucose uptake (**I**), lactate (**J**), ATP (**K**) and NADPH (**L**) production were determined as described in Methods.

(M) and (N) ECAR (**M**) and OCR (**N**) were determined as described in Methods.

Figure S5. The effect of SOX8 and FOXK1 on chemoresistance and cell senescence in ovarian cancer.

(A) Immunoblotting analysis of p-SOX8 (Ser327).

(B) IC50 of cisplatin.

(C) Percentage of apoptotic cells. Cells were treated with 5 mg/L cisplatin for 48h.

(D), (E) and (F) Immunoblotting analysis of SOX8 and FOXK1.

(G) Cell viability tested by CCK-8 assay. *, $P < 0.05$, **, $P < 0.01$.

(H) IC50 of cisplatin.

(I) Percentage of apoptotic cells. Cells were treated with 5 mg/L cisplatin for 48h.

(J) Percentage of β-galactosidase staining in ovarian cancer cells.

(K) Heat map of genes associated with cell senescence and chemoresistance.

(L) and (M) P16 (**L**) and hTERT (**M**) promoter activity affected by SOX8 and FOXK1. **, $P < 0.01$.

(N) ChIP results of the binding of FOXK1 to the promoter of P16 and hTERT.

Figure S6. SOX8 and FOXK1 modulates the glucose metabolism in ovarian cancer cells.

(A), (B), (C) and (D) Glucose uptake (**A**), ATP (**B**), lactate (**C**) and NADPH (**D**) production were determined as described in Methods.

(E) and (F) ECAR (**E**) and OCR (**F**) were determined as described in Methods.

(G) Heat map of genes associated with glucose metabolism.

(H) and (I) HK2 (**H**) and LDHA (**I**) promoter activity affected by SOX8 and FOXK1.

(J) ChIP results of the binding of FOXK1 to the promoter of HK2 and LDHA.

Figure S7. Aurora-A affected SOX8 transcription by regulating c-Myc and modulated the expression of proteins associated with cell senescence and glycolysis in tumor tissues.

(A) c-Myc expression was tested by immunoblotting assay. Aurora-A silencing reduced the expression level of oncogenic transcription factor c-Myc.

(B) SOX8 mRNA expression level was tested by qRT-PCR. C-Myc overexpression significantly reversed Aurora-A silencing induced low mRNA expression of SOX8.

(C) SOX8 promoter activity affected by Aurora-A knockdown and c-Myc overexpression. C-Myc overexpression significantly reversed Aurora-A silencing induced low transcription of SOX8.

(D) Relation between Aurora-A and hTERT.

(E) Relation between Aurora-A and P16.

(F) Relation between Aurora-A and HK2.

(G) Relation between Aurora-A and LDHA.

Figure S8. Survival analysis and the immunofluorescence assay of Aurora-A and proteins related to cell senescence and glycolysis.

(A) and (B) Overall survival of cisplatin-sensitive and -resistant ovarian cancer patients with different expression levels of Aurora-A, SOX8 and FOXK1.

(C) and (D) Immunofluorescence of Aurora-A and proteins associated with cell senescence and glycolysis in cisplatin-sensitive and -resistant ovarian cancer patients.

Supplementary Table 1. The correlation of clinicopathological characteristics and overall survival in ovarian cancer patients

Prognostic factors	Patients N (%)	Univariate		Multivariate
		P	HR (95 % CI)	P
**All patients**	431 (100)			
Age (years)				
≤65 (median)	242 (56.15)			
> 65 (median)	189 (43.85)	0.113	1.901 (1.106-2.765)	0.790
**FIGO Stage**				
Ia ~ IIIb	120 (27.84)			
IIIc ~ IV	311(72.16)	**0.000**	2.998 (1.697-3.987)	**0.004**
**Grade**				
Low	105 (24.36)			
Moderate ~ High	326 (75.64)	0.081	3.105 (1.241-4.507)	0.312
**Histology**				
Serous	343 (79.58)			
Other	88 (20.42)	0.072	3.811 (1.021-6.231)	0.218
**Diameter**				
≤8cm	269 （62.41）			
>8cm	162 （37.59）	0.198	2.315 (1.543-3.960)	0.301
**Lymph node**				
Positive	274 (63.57)			
negative	157 (36.43)	0.056	2.674 (1.671-4.072)	0.098
**Ascite**				
Positive	291 (67.52)			
negative	140 (32.48)	0.363	1.470 (0.965-2.856)	0.651

Kanplan-Meier survival analysis and Cox proportional hazards regression analysis.

Supplementary Table 2. The primary antibodies

Primary antibodies	Company (NO.)
Aurora-A	Abcam (ab13824)
SOX8	Abcam (ab221053); Santa Cruz Biotechnology (sc-374446)
p-SOX8(Ser327)	WanleiBio (WL04464)
FOXK1	Abcam (ab18196)
β-actin	Cell signaling technology (4970)
P16	Sigma Aldrich (MAB4133); Abcam (ab51243)
P53	Abcam (ab32389)
p-P53(Ser315)	Cell signaling technology (2528)
P21	Abcam (ab109199)
p-Rb (Ser780)	Cell signaling technology (9307)
AKT	Cell signaling technology (9272)
p-AKT(Ser473)	Cell signaling technology (4060)
hTERT	Abcam (ab230527)
LDHA	Abcam (ab125683)
GLUT1	Cell signaling technology (12939)
HK2	Abcam (ab104836)
SIRT1	Abcam (ab110304)
Histone 3	Cell signaling technology (9715)
p- Aurora-A(Thr288)	Cell signaling technology (3079)

Supplementary Table 3. The primer sequences of qRT-PCR assay

Gene	Number	Primer
Aurora-A	F	TGAATAACACCCAAAAGAGCAAG
	R	ACTTTCCTTTACCCAGAGGGC
SOX8	F	CCAGAACATCGACTTCAGCAAC
	R	ACTGGTCGAACTCGTGGACG
FOXK1	F	AGCAGTGTACCTTCCGGTTTC
	R	GTGGATCTTCAGAGGGGAGATC
P16	F	TCCAGGTCATGATGATGGGC
	R	CATCTATGCGGGCATGGTTAC
Caspase-3	F	CACCTGGTTATTATTCTTGGCG
	R	CGGTTAACCCGGGTAAGAAT
P53	F	AACTGCGGGACGAGACAGA
	R	AGCTTCAAGAGCGACAAGTT
P21	F	CTGTCTTGTACCCTTGTGCCTC
	R	TCCTCTTGGAGAAGATCAGCC
P38	F	AGTTGATATTTGGTCAGTGGGATG
	R	TCGGCATCTGAGTCAAAGACTG
Rb	F	CGCTCTTGAGGTTGTAATGGC
	R	TCAAGTTGCCTTCTGCTTTGATA
HK2	F	GATTGTCCGTAACATTCTCATCGA
	R	TGTCTTGAGCCGCTCTGAGAT
H2AX	F	CTGGAGTACCTCACCGCTGAG
	R	TGTTGAGCTCCTCGTCGTTG
AKT	F	TTCTATGGCGCTGAGATTGTGT
	R	GCCGTAGTCATTGTCCTCCAG

Gene	Number	Primer
P65	F	CGCATCCAGACCAACAACA
	R	TGCCAGAGTTTCGGTTCAC
CDK1	F	CTACAGGTCAAGTGGTAGCCATG
	R	TATAACCTGGAATCCTGCATAAGC
ATM	F	GCGTGCCAGAATGTGAACAC
	R	GCCAATACTGGACTGGTGCTT
Ras	F	GTAGGCAAGAGTGCCTTGACG
	R	ACACAAAGAAAGCCCTCCCC
hTERT	F	CCGATTGTGAACATGGACTACG
	R	AGCACGCTGAACAGTGCCTT
GLUT1	F	CTTTGTGGCCTTCTTTGAAGT
	R	CCACACAGTTGCTCCACAT
GLUT4	F	TGGAAGGAAAAGGGCCATGCTG
	R	CAATGAGGAATCGTCCAAGGATG
LDHA	F	TGGAGATTCCAGTGTGCCTGTATGG
	R	CACCTCATAAGCACTCTCAACCACC
ALDOA	F	GTTATCAAATCCAAGGGCGGTGTT
	R	AGTCAGCTCCGTCCTTCTTGTAC
ALDOB	F	CACCATTCAAGGGCTTGATGGCCT
	R	TTCCTGGATAGCGAGGCTGGAT
PGK1	F	CAAGGTTAAAGCCGAGCCAGCCAA
	R	GCCTTCTGTGGCAGATTGACTCC
PFKL	F	CACAGGTGCCAACATCTTCCGCA
	R	TCATGTCGGTGCCGCAGAAGTCG

Gene	Number	Primer
PFKP	F	AGAGGACCTTCGTTCTGGAGGT
	R	GGGCACGGTTCTCCGAGAGTTT
G6P	F	GGTACACAGGCAAGACCATC
	R	GTTTTGGCAATGTGAGTTCC
ENO1	F	GCTCCGGGACAATGATAAGACTCG
	R	CTGTTCCATCCATCTCGATCATC
SIRT1	F	TGCTGGCCTAATAGAGTGGCAAAG
	R	TCTGGCATGTCCCACTATCACTGT
SIRT2	F	GGAGGCATGGACTTTGACTCCAAG
	R	CATCTATGCTGGCGTGCTCCCT
SIRT4	F	ATGTGGATGCTTTGCACACCAAGG
	R	TTCAGGACTTGGAAACGCTCTTGC
SIRT6	F	AGCAGGAACGCCGACCTGTCCAT
	R	TCAGCATGGCGGTCGTGCTTGGT
GAPDH	F	GCACCGTCAAGGCTGAGAAC
	R	TGGTGAAGACGCCAGTGGA

Supplementary Table 4. Aurora-A binding proteins

	Aurora-A-binding proteins
**1**	Serum albumin
**2**	Putative elongation factor 1-alpha-like 3
**3**	Keratin, type II cytoskeletal 75
**4**	Keratin, type II cytoskeletal 6B
**5**	Keratin, type I cytoskeletal 10
**6**	Keratin, type I cytoskeletal 14
**7**	Partitioning defective 6 homolog gamma
**8**	60S ribosomal protein L4
**9**	Immunoglobulin heavy constant gamma 1
**10**	Tubulin gamma-1 chain
**11**	Protein piccolo
**12**	ATP synthase subunit beta, mitochondrial
**13**	Keratin, type I cytoskeletal 9
**14**	E3 ubiquitin-protein ligase TRIM4
**15**	Probable guanine nucleotide exchange factor MCF2L2
**16**	Protein Mdm4
**17**	tRNA wybutosine-synthesizing protein 3 homolog
**18**	40S ribosomal protein S9
**19**	Ubiquitin carboxyl-terminal hydrolase 7
**20**	Carboxyl-terminal PDZ ligand of neuronal nitric oxide synthase protein
**21**	Hexokinase-1
**22**	Protein bassoon
**23**	Cyclic AMP-dependent transcription factor ATF-6 alpha
**24**	Armadillo-like helical domain-containing protein 3
**25**	Transcriptional regulator ATRX
**26**	Transcription factor SOX-8
**27**	Sorting nexin-2
**28**	LisH domain-containing protein ARMC9
**29**	1,5-anhydro-D-fructose reductase
**30**	Fibronectin type III domain-containing protein 1
**31**	Complement factor D
**32**	Peptidyl-prolyl cis-trans isomerase FKBP9
**33**	Canalicular multispecific organic anion transporter 1
**34**	Probable E3 ubiquitin-protein ligase HECTD4
**35**	Zinc transporter 7
**36**	Synaptic vesicle membrane protein VAT-1 homolog
**37**	Serine/threonine-protein kinase SMG1
**38**	Polycystic kidney disease protein 1-like 2

**39**	Rhomboid-related protein 3
**40**	Uncharacterized protein C22orf42
**41**	Fibronectin type III and SPRY domain-containing protein 2
**42**	Izumo sperm-egg fusion protein 3
**43**	Pogo transposable element with ZNF domain
**44**	Inversin
**45**	Protein ZGRF1
**46**	DNA replication factor Cdt1
**47**	Zinc finger protein 268
